# Genome Analyses Reveal Diverse Riverine Genetic Contributions to the Lake Malawi Cichlid Radiation

**DOI:** 10.1111/mec.17786

**Published:** 2025-06-10

**Authors:** Sophie Gresham, Bosco Rusuwa, Maxon Ngochera, George F. Turner, Martin J. Genner, Milan Malinsky, Hannes Svardal

**Affiliations:** ^1^ Evolutionary Ecology Group, Department of Biology University of Antwerp Antwerp Belgium; ^2^ Department of Biological Sciences Chancellor College, University of Malawi Zomba Malawi; ^3^ Department of Fisheries Ministry of Natural Resources & Climate Change Lilongwe Malawi; ^4^ School of Natural Sciences Bangor University Bangor UK; ^5^ School of Biological Sciences University of Bristol Bristol UK; ^6^ Department of Biology Institute of Ecology and Evolution, University of Bern Bern Switzerland; ^7^ Naturalis Biodiversity Centre Leiden the Netherlands

**Keywords:** ABBA‐BABA, adaptive radiation, hybridization, introgression, Lake Malawi cichlid

## Abstract

Comparative studies of whole genomes have increasingly shown that genetic introgression between closely related species is surprisingly common across the tree of life, making the description of biodiversity and understanding the process of speciation complex and challenging. The adaptive radiation of cichlid fishes in Lake Malawi, that is characterised by hybrid origins and cases of recent introgression, provides a valuable model system to study the evolutionary implications of introgression. However, many potential sources of introgression into the radiation have not yet been investigated. Here we use whole genome data from 239 species from Lake Malawi and 76 species from surrounding African river and lake systems to identify previously unknown introgression events involving the Malawi radiation. Computing genome‐wide excess allele sharing (ABBA‐BABA statistics) and window‐based statistics, we find that three independent riverine cichlid lineages show significantly higher allele sharing with the Malawi radiation than expected, suggesting historical genetic exchange. Introgressed haplotypes are distributed relatively uniformly across the Malawi radiation, indicating that most hybrid‐derived polymorphism was acquired and sorted before the formation of the contemporary Malawi radiation. Our results point towards several previously unknown contributors to the Malawi cichlid hybrid swarm and show that the history of one of the largest vertebrate radiations is more complex than previously thought.

## 1 Introduction

Over the last two decades, a growing number of genome‐comparison studies have revealed that hybridization between closely related species is surprisingly common across the tree of life (Martin et al. 2013; Lamichhaney et al. 2015; Mallet et al. 2016; Novikova et al. 2016). However, the effect and significance of this phenomenon in the evolution of biodiversity—specifically the formation and maintenance of new species—is still debated. On the one hand, gene flow can prevent speciation by impeding the divergence of evolutionary lineages or even lead to their collapse (Mayr 1963). On the other hand, gene flow can facilitate the spread of beneficial alleles among lineages, thereby promoting ecological adaptation and accelerating speciation (Seehausen 2004).

Adaptive radiations, formed by the rapid diversification of one or more ancestral lineages into many ecologically diverse species, are suggested to account for a considerable proportion of extant biodiversity (Berner and Salzburger 2015; Wiens 2024). As such, identifying the key mechanisms driving adaptive radiation is important for understanding the evolution of biodiversity. Since adaptive radiations can involve many rapid ecological adaptation and speciation events, the sharing of genetic variation through gene flow may be particularly important. This is because combinatorial mixing of existing alleles represents an available source of functional genetic variation, overcoming the limits to adaptation imposed by the comparatively slow arrival of new mutations (Wagner et al. 2012; Brawand et al. 2014; Marques et al. 2019).

African cichlid fishes are particularly well known for their ecologically and phenotypically diverse adaptive radiations, having generated hundreds of species over very short evolutionary timescales in Lake Victoria (and neighbouring lakes Albert, Edward and Kivu), Lake Tanganyika and Lake Malawi (Kocher 2004; Salzburger 2018; Svardal et al. 2021). Approximately 80% of all African cichlid species belong to the strikingly diverse pseudocrenilabrine group (tribe Pseudocrenilabrini, formerly Haplochromini), which dominates the cichlid faunas of East African lakes and rivers, and represents around 6% of all freshwater fish species diversity (Fricke et al. 2024). Consistent with the role of gene flow in promoting diversification, studies have shown that the major pseudocrenilabrine radiations representing most of this diversity—those of the Lake Victoria Region (~500 species), Lake Tanganyika (40 Tropheina species) and Lake Malawi (~800 species)—have all been fuelled by hybridization and genetic introgression from riverine lineages before or soon after their appearance (Meier et al. 2017, 2023; Meyer et al. 2017; Irisarri et al. 2018; Svardal et al. 2020; Ronco et al. 2021). Impressively, each of these radiations has independently evolved similar ecotypes following similar stages of trophical diversification (Kocher et al. 1993; Salzburger 2009, 2018). This contrasts with the pseudocrenilabrine riverine lineages, which are relatively species‐poor, and show a limited number of distinct ecotypes (Seehausen 2015). Despite differences in diversity, many riverine and lacustrine ecotypes overlap, which could partially explain the successful transport of genetic material from river to lake environments through hybridization (Seehausen 2015). Importantly, hybridization also has the capacity to generate novel lake phenotypes through the process of transgressive segregation—the formation of extreme phenotypes in hybrids not observed in parental species (Bell and Travis 2005; Stelkens and Seehausen 2009; Holzman and Hulsey 2017).

In addition to ancient introgression at the base of radiations, recent gene flow into specific lineages within radiations has contributed to generating the high diversity of ecotypes seen in radiations, by providing genetic material needed for lineage‐specific ecological adaptations (Meier et al. 2023). For pseudocrenilabrines, which have retained the potential for hybridization between phylogenetically distant species (Stelkens et al. 2010; Loh et al. 2013), potentially many lineages could have genetically contributed at the base of radiations or to specific lineages. However, most previous genomic studies have focused on specific lake radiations, missing a geographically wider context of lineages that could have contributed genetic diversity to these radiations. This means that our understanding of the full history of introgression into pseudocrenilabrine radiations may be incomplete.

In Lake Malawi more than 800 cichlid species have originated within the last 800 thousand years from a common ancestral population, making it the largest recent vertebrate adaptive radiation (Turner et al. 2001; Ivory et al. 2016; Malinsky and Salzburger 2016). Svardal et al. (2020) demonstrated that an ancestral ‘hybrid swarm’ of Malawi cichlids contained a mixture of two divergent ancestries, related to two distinct extant pseudocrenilabrine cichlid lineages: (1) the ‘Victoria group’ lineage, consisting of the adaptive radiations in Lakes Victoria, Albert, Edward and Kivu (also known as the Lake Victoria Region Superflock; LVRS), as well as several riverine pseudocrenilabrines, and (2) *Astatotilapia* sp. ‘Ruaha blue’, a riverine pseudocrenilabrine from the Rufiji River catchment in Tanzania, where its current range overlaps with members of the Victoria group (Genner et al. 2015; Turner et al. 2019). Svardal et al. (2020) showed that Lake Malawi cichlids have similar proportions of ‘Victoria‐like’ and ‘Ruaha‐blue‐like’ ancestry, persisting in fixed and relatively long genomic stretches, suggesting that most hybridization‐derived polymorphism was sorted at the haplotype level before the start of the radiation. At the same time, single nucleotide polymorphisms (SNPs) with hybridization‐derived origin have diverged more strongly between Malawi cichlid ecomorphological groups (i.e., distinct lineages within the Malawi radiation that differ in ecology and morphology) compared to other SNPs and neutral simulations. This suggests that hybridization‐derived polymorphisms have been under selection, and contributed to adaptive divergence in the early stages of the radiation (Svardal et al. 2020). This phenomenon has also been reported as contributing to the hybrid origins of the independent LVRS cichlid radiations (Meier et al. 2017).

Whilst Svardal et al. (2020) demonstrated a hybrid origin of the Malawi radiation with two contributing lineages, the study was limited to 13 whole genome samples of Malawi cichlids and a phylogenetically relatively narrow selection of non‐Malawi cichlid species (13 species from the Victoria group, and three further outgroup species/lineages). Therefore, it is currently not clear whether there have been additional contributions from other pseudocrenilabrine cichlid lineages, either to the ancestral Lake Malawi cichlid hybrid swarm or to specific lineages of the radiation. Furthermore, the study by Svardal et al. (2020) was based on an alignment to a reference genome of the distantly related Nile tilapia ( *Oreochromis niloticus* ), effectively limiting the analysis to 32% of the genome, potentially leading to a systematic underdetection of actual genomic diversity.

In this study, we analysed whole genome sequence data from a large and representative set of Lake Malawi cichlids (500 samples from 239 species) alongside a comprehensive sample of non‐Malawi pseudocrenilabrine cichlid species (111 samples from 76 species), many of which are from taxonomic groups that have not been considered in previous gene flow analyses. We also took advantage of a newly available high‐quality reference genome of the Malawi cichlid *Astatotilapia calliptera* (Rhie et al. 2021), enabling us to analyse a much larger proportion of the genome at higher alignment qualities. Using a combination of genome‐wide and window‐based approaches, we asked whether there were additional contributions to the Malawi cichlid ancestral population, or to specific lineages within the Malawi cichlid adaptive radiation.

## 2 Material and Methods

### 2.1 Sample Selection, Sequence Alignment, Variant Calling and Filtering

Illumina short‐read sequence data for all samples were acquired from the Sequence Read Archive (Table S1 for list of BioProjects and BioSamples). For the Malawi radiation, we selected a maximum of two samples per species from each of the seven main Malawi ecomorphological groups, resulting in 13 *Diplotaxodon* species (subtribe Rhamphochromina), 12 *Rhamphochromis* species (subtribe Rhamphochromina), 102 shallow benthic species (subtribe Cyrtocarina), 37 deep benthic species (subtribe Cyrtocarina), 10 ‘utaka’ species (subtribe Cyrtocarina) and 64 ‘mbuna’ species (subtribe Pseudotropheina). For the seventh ecomorphological group, consisting of the single species *Astatotilapia calliptera* that is present in both the main lake and proximate rivers, we selected 113 samples from twelve populations. In total, the analysed data comprised 500 samples from the Malawi radiation, belonging to 239 species, representing approximately 30% of the estimated cichlid species diversity within Lake Malawi. For non‐Malawi cichlid species, we selected eleven representative species from the Lake Tanganyika pseudocrenilabrine radiation, 41 species from ‘Victoria group’ (consisting of the Lake Victoria Region Superflock radiations and their riverine sister lineages), and 20 species from smaller lake and riverine lineages within the Zambezi Basin, Congo Basin, Nile Basin and the East Central Coast region. All non‐Malawi taxonomic names herein follow 'Eschmeyer's Catalog of Fishes' (Fricke et al. 2024). We note that whilst *Astatotilapia bloyeti* has been identified as a synonym of *Haplochromis paludinosus* (Turner et al. 2021), we consider these species separately for analyses presented here.

Sequence data were aligned to the high‐quality *Astatotilapia calliptera* fAstCal1.2 reference genome (NCBI GenBank: GCA_900246225.3) using BWA‐MEM version 0.7.17‐r1188 (Li 2013). Variant calling was carried out using BCFtools version 1.14 (Li 2011). We filtered out sites with an overall mapping quality (MQ) of < 50, sites where > 10% of mapped reads have a MQ = 0, sites for which the MQ significantly differed between the forward and reverse strands (*p* < 0.001 in a Mann–Whitney *U* test), sites with a *p*‐value of excess heterozygosity < 0.2, and sites with > 20% missing genotypes. Additionally, we removed sites which showed abnormally high sequencing depth across all samples (> 1.5 standard deviation) and heterozygous sites which had a significantly biased read depth of the reference and alternative alleles (PHRED score > 20 in a binomial test). For any two filtered sites which were within 10 bp of each other, we also removed any other sites in between. Only biallelic SNPs were kept for further analyses. Six large inversion regions (chr2:9800000–32,540,000, chr9:12000000–28,900,000, chr10: 11300000–29,320,000, chr11:6789301–28,721,782, chr13:9780000–29,600,000, chr20:2157743–4,792,622) as identified by Blumer et al. (2025) were removed for all analyses. Finally, we added to the filtered VCF files a reconstructed sequence of the common ancestor between the Cyphotilapiini and Pseudocrenilabrini tribes as an additional sample, here referred to as the ‘ancestral state’ sample (for details see Blumer et al. (2025) materials and methods ‘ancestral state inference’).

### 2.2 Phylogenetic Tree Inference and Species Grouping

To infer the phylogenetic relationships, we reconstructed and compared phylogenetic trees created using neighbour‐joining (NJ) and maximum likelihood (ML) methods. For the NJ method, we calculated pairwise SNP distance matrices in 100 kb non‐overlapping windows along the genome using the pypopgen3 diversity module (https://github.com/feilchenfeldt/pypopgen3/blob/master/modules/diversity.py). Using the same module, we computed a genome‐wide consensus NJ tree by summing distance matrices across all windows and computing a tree using the ancestral sample set as an outgroup. To assess the robustness of species relationships within the genome‐wide NJ tree, we used a block‐bootstrapping approach by resampling windows with replacement. Node support was determined by evaluating whether clades in the original tree were recovered in each bootstrap replicate. Node support values give the proportion of replicates which support the node. To create ML trees, we used the GTR + G + I model in IQ‐TREE version 2.2.0‐beta (Nguyen et al. 2015) to calculate trees in 100 KB non‐overlapping windows along the genome, which were then concatenated into a single ‘tre’ file and then built into a consensus tree using ASTRAL‐III version 5.15.5 (Zhang et al. 2018). The ancestral state sample was used as an outgroup in IQ‐tree. Input PHYLIP files used for IQ‐TREE were converted from VCF files using the vcf2phylip.py script (available at https://github.com/edgardomortiz/vcf2phylip). Both the NJ and ML trees were rooted with the ancestral state sample. Trees were visualised using FigTree version 1.4.4 (http://tree.bio.ed.ac.uk/software/figtree/), and using these trees, we assigned all non‐Malawi species into monophyletic groups. Non‐Malawi species groupings were checked and named in reference to those already described in the literature.

### 2.3 Genome‐Wide Tests for Excess Allele Sharing

To detect potential cases of gene flow between Malawi and non‐Malawi species, we computed the ABBA‐BABA statistics, namely Patterson's D and f_4_‐ratio, which are widely used in admixture analyses (Durand et al. 2011; Patterson et al. 2012). These statistics test for genome‐wide excess allele sharing in four taxon trees (consisting of species P1, P2, P3 and O, the outgroup), revealing patterns of introgression even among highly diverged lineages (Martin et al. 2015). We calculated statistics using a custom‐built Snakemake workflow, ‘Dtrios Cluster’ (https://github.com/feilchenfeldt/dtools/tree/master/dtrios_cluster), which parallelises the Dsuite Dtrios software by splitting the chromosomes and the list of species trios into ‘chunks’ (Malinsky et al. 2021). Dtrios Cluster calculates ABBA‐BABA statistics for all possible combinations of species as P1, P2 and P3 where the species relationships matches the topology (((P1, P2), P3), O). Dtrios Cluster was implemented on all Malawi and non‐Malawi species, with the ancestral state sample set as the outgroup species. For each of the resultant tests (also referred to as trios), Dtrios Cluster outputs the P1, P2 and P3 species, the D‐statistic, *Z*‐score, *p*‐value, f_4_‐ratio and the count of BBAA, ABBA and BABA sites in which the statistics are calculated. Positive D statistics indicate excess allele sharing of derived alleles between species P2 and P3, which may then be explained by recent gene flow between P2 and P3 which doesn't affect P1. To detect potential cases of gene flow between target species, we filtered specific combinations of species as P1, P2 and P3 from the output Dtrios Cluster file (containing all possible test trios). To account for multiple testing, after filtering species P1, P2 and P3, we divided the *p*‐value threshold (0.05) by the total number of trios (i.e., the Bonferroni method), resulting in a highly conservative *p*‐value threshold. We selected significant trios as those with a *p*‐value below this threshold.

To test for excess allele sharing between *Astatotilapia gigliolii* and specific Malawi *Astatotilapia calliptera* populations, we carried out a new run of Dtrios Cluster with a different set of species. We tested the five *A. gigliolii* individuals and 19 *A. calliptera* populations, set as separate species, and included representative species from the Victoria group ( *Astatotilapia flaviijosephi* ) and the Malawi group ( *Labeotropheus fuelleborni* , *Copadichromis chrysonotus* , *Diplotaxodon limnothrissa* , *Mylochromis subocularis*, *Alticorpus peterdaviesi* and *Rhamphochromis woodi* ). *Astatotilapia flaviijosephi* was chosen as the Victoria group representative species because it is the only Victoria species in our analyses that is outgroup to the Eastern and Upper Nile lineages (*Astatotilapia bloyeti, Haplochromis paludinosus, Haplochromis gracilior* and *Haplochromis pharyngalis* ) that are the genomic contributors to the LVRS (Meier et al. 2017), and therefore will escape the potential confounding effects of within‐Victoria gene flow. Again, we filtered the resulting tests for specific combinations of P1, P2 and P3 species, and applied multiple testing correction on *p*‐values of subsequent filtered tests. All test trios were analysed and results plotted in R.

### 2.4 Lineage Split Time Estimation

We estimated split times between lineage pairs based on sequence divergence per accessible site between groups. To calculate sequence divergence per accessible site, we computed a pairwise SNP distance matrix, including the Malawi and non‐Malawi samples and the ancestral state sample, for each chromosomal VCF using PLINK version v1.90b6.21 (Purcell et al. 2007). We summed all distance matrices for all chromosomes and then divided by the total size of ‘accessible genome’ (i.e., the unmasked genome). We calculated the net nucleotide divergence (D_A_) between lineages *x* and *y* as:

(1) $$D_{A}=\text{median between group}\;d_{\mathrm{xy}}-\text{mean within group heterozygosity}$$

where the between group d_xy_ is the median cross‐lineage pairwise sequence divergence per accessible site, and the mean within group heterozygosity is the mean of the median group x and median group y proportion of heterozygous sites per accessible site (total heterozygosity divided by the accessible genome size). Heterozygosity was calculated using vcftools v0.1.16 (Danecek et al. 2011). To estimate uncertainty in D_A_ we used a chromosome‐based jackknife resampling approach: we recalculated both the median between group d_xy_ and mean within group heterozygosity by leaving out one chromosome at a time. We calculated the upper and lower D_A_ estimates using ± 3 jackknife standard deviations with:

(2) $$D_{A}\;\left(\text{upper}/\text{lower}\right)=\text{mean}\left(D_{A}\right)\pm 3∙\sqrt{\frac{1}{n}\mathrm{Var}\left(D_{A}\right)}$$

where n is the number of chromosomes (22). To create more conservative intervals and also capture the variation in the processes that determine D_A_, we removed Bessel's correction (1/n) from the jackknifing variance formula. The upper and lower estimated split times (in years) between lineages *x* and *y* were calculated as:

(3) $$\text{Split time}\;\left(\text{upper}/\text{lower}\right)=\frac{D_{A}\left(\text{upper}/\text{lower}\right)∙\text{generation time}}{2\mu }$$

using a generation time of 3 years and a per site mutation rate (μ) of 3.5 × 10^−9^ per generation (Malinsky et al. 2018). We note that variation in generation time and mutation rate will also affect split time estimates, but these are not accounted for in our estimates. However, since our purpose of calculating split times between lineages is to compare them against each other rather than to generate accurate timings, variation in mutation rate over time is inconsequential, assuming that variation in mutation rates has affected all lineages equally.

### 2.5 Principal Component Analysis and ADMIXTURE

To investigate very recent cases of hybridization between *A. gigliolii* and *A. calliptera* in the Rovuma catchment, we carried out a Principal Component Analysis (PCA) using PLINK v1.90b6.21 (Purcell et al. 2007), followed by an ADMIXTURE (Alexander et al. 2009) analysis, using five *A. gigliolii* samples, four *A. calliptera* Rovuma samples and 15 *A. calliptera* non‐Rovuma samples. To reduce the influence of linkage disequilibrium for both analyses, we pruned the callset using PLINK in sliding windows of 50 SNPs with a step of 10 SNPs and a r^2^ threshold of 0.1.

### 2.6 Tests for Excess Allele Sharing in Windows

**FIGURE 1:**
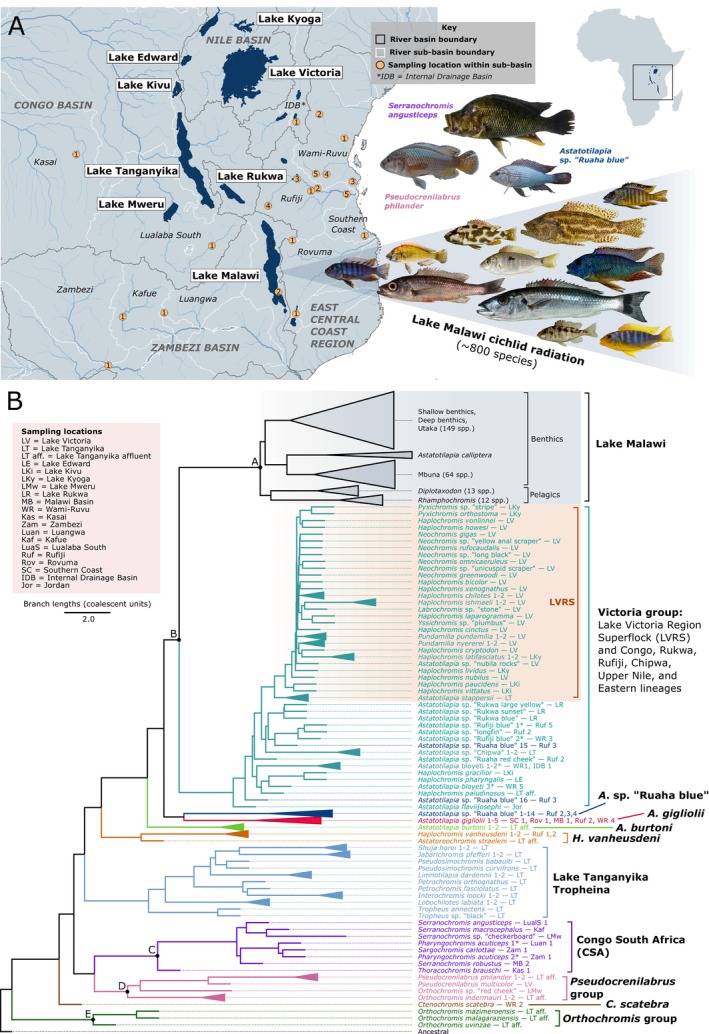
Phylogenetic reconstruction of African pseudocrenilabrine cichlids. (A) Sampling map of East African lakes and their drainage basins. Basins are outlined in white with a solid line and sub‐basins are outlined in white with a dotted line. For samples collected from rivers or small water bodies, sampling points are shown and numbered within each sub‐basin (e.g., Rovuma 1 is the first location within the Rovuma sub‐basin). The central Jordan river system, the sampling location of *Astatotilapia flaviijosephi* , is not shown. Photos by Luka Moritz Blumer ( *Astatotilapia calliptera* ), Alex Hooft van Huysduynen/Hannes Svardal/Ilia Artiushin/Valentina Burskaia (all other Malawi species), George Turner (*Astatotilapia* sp. ‘Ruaha blue’), Robert Taylor ( *Pseudocrenilabrus philander* , available at http://specify‐attachments‐saiab.saiab.ac.za/originals/sp6‐3284146409238926730.att.JPG, licensed under CC by 4.0), and the South African Institute for Aquatic Biodiversity ( *Serranochromis angusticeps* , available at https://www.inaturalist.org/photos/97168187, licensed under CC by 4.0). (B) A maximum likelihood phylogenomic tree for Malawi and non‐Malawi African pseudocrenilabrine cichlids (*n* = 612), rooted to a reconstructed ancestral state sample (see methods). The scale bar gives genomic distance in coalescent units and is based on the amount of discordance in gene trees. The Malawi radiation (highlighted in grey), containing 239 species in our callset, has been visually collapsed into five major groups, each comprising of the ecomorphological groups described by Malinsky et al. (2018). The ecomorphological groups have been approximately sized according to the number of species in each group. Non‐Malawi species were assigned to 10 phylogenetic groups and members are coloured according to their group membership. The Lake Victoria Region Superflock (LVRS) sub‐group of the Victoria group is highlighted in orange. Sampling locations for each sample are given after a dash. Samples which were collected from major lakes (or affluents of major lakes) are labelled with the lake name. All other samples are labelled with the sub‐basin name and location number which corresponds to the map in a. Nodes A‐E mark split times for the Malawi radiation (estimated 1.17–1.19 Mya), the Malawi/Victoria clade (4.56–4.65 Mya), the CSA group (5.12–5.18 Mya), the *Pseudocrenilabrus* group (6.99–7.06 Mya), and the *Orthochromis* group (1.62–1.66 Mya) respectively.

To complement genome‐wide gene flow tests, we tested for local signals of gene flow along the genome. We used the Dsuite module Dinvestigate to calculate the *f*_dM_ statistic (Malinsky et al. 2015) in 50 SNP non‐overlapping windows across all chromosomes. Like the D statistic, the *f*_dM_ statistic simultaneously tests for excess allele sharing between species P3 and P1 (negative values) and species P3 and P2 (positive values). Under the null hypothesis of no gene flow between species P3 and P1 or P2, the distribution of *f*_dM_ values across all windows will be normally distributed around 0. For all Dinvestigate tests, we combined all Victoria group species and all Malawi species as single ‘species’ as P1 and P2, respectively. As P3, we tested the five non‐Malawi groups which showed excess allele sharing with the Malawi radiation in the previous genome‐wide ABBA‐BABA tests (*Astatotilapia* sp. ‘Ruaha blue’, *A. gigliolii*, CSA, *Pseudocrenilabrus*, *Orthochromis*; Figure 1). As with the P1 and P2 species, we combined all species from each P3 group into one test ‘species’. Thus, in total, we ran five sets of test trios, each with P1 = Victoria, P2 = Malawi and P3 = *A*. sp. ‘Ruaha blue’, *A. gigliolii*, CSA, *Pseudocrenilabrus* or *Orthochromis*.

For each P3 non‐Malawi group tested, we selected outlier windows with a significantly positive *f*_dM_ value as candidate windows for gene flow between species P2 and P3. Under the null hypothesis of no gene flow, outlier *f*_dM_ values are expected on both sides of the normal distribution of *f*_dM_ values across all windows. Therefore, to select outlier windows with meaningfully positive *f*_dM_ values, we used the absolute value of the *f*_dM_ value marking the boundary for the most negative 0.5% of windows as a threshold for significantly positive windows. Because *f*_dM_ windows are defined on a set SNP basis rather than set base pair sizes, in some cases this leads to large, fragmented windows which are mostly made up of large sections of inaccessible genome. To prevent this, we removed *f*_dM_ windows larger than 20 kb.

## 3 Results

### 3.1 Phylogenomic Context of the Lake Malawi Radiation Among East African Pseudocrenilabrine Cichlids

Alignment of whole genome short read sequencing data from 612 individuals (mean 20x and minimum 6x coverage) and variant calling yielded 147 million biallelic SNPs. Of these, 103 million SNPs (70.5%) passed quality filtering; 35 million were polymorphic only within the Lake Malawi radiation, whilst 45 million were polymorphic only outside of Malawi or had fixed differences between Malawi and non‐Malawi species.

The phylogenomic trees built using maximum likelihood (ML; Figure 1B) and neighbour‐joining (NJ; Figure S1) methods supported the monophyly of the Malawi radiation with respect to other African pseudocrenilabrine cichlid lineages. In line with previous studies, the Victoria group, consisting of the Lake Victoria Region Superflock (LVRS) and a geographically widespread assemblage of riverine and lacustrine *Astatotilapia/Haplochromis* spp. including the Upper Nile and Congolese lineages sensu (Meier et al. 2017), formed a sister group to the Malawi radiation. The ML tree enabled placement of non‐Malawi species into ten monophyletic groups (Figure 1B, Table S1), which all had an ASTRAL local posterior probability (LPP) value of 1. Some nodes within the LVRS clade and the *Astatotilapia* sp. ‘Ruaha blue’ clade had LPP values below 1 (Figure S2).

In some cases, phylogenetic placement was inconsistent with morphological taxonomic assignment. Most notably, while most *A*. sp. ‘Ruaha blue’ samples clustered in a sister‐relationship to *Astatotilapia gigliolii* (Svardal et al. 2020), two specimens clustered within the Victoria group in both ML and NJ trees. Their placement varied, either relatively basal, or together with Victoria species from the same river catchment. Computing f_4_‐ratios for these aberrant samples confirmed that they were admixed between *A*. sp. ‘Ruaha blue’ and a Victoria group lineage—most likely *Astatotilapia* sp. ‘Ruaha red cheek’ (Figure S3). Given our focus on gene flow involving the Lake Malawi radiation, we excluded these two *A*. sp. ‘Ruaha blue’ specimens from further analyses. Four other non‐Malawi species were non‐monophyletic in either one or both trees (asterisks in Figure 1B and Figure S1). To enable further analyses, the two specimens each of *Haplochromis chilotes* , *Pharyngochromis acuticeps* and *Astatotilapia*. sp. ‘Rufiji blue’ were treated as separate taxonomic entities (named 1 and 2) in subsequent analyses. Meanwhile the abnormally clustering sample of *Astatotilapia bloyeti* was excluded. The few cases of non‐monophyletic Malawi species were not excluded from further analyses (Figure S4).

### 3.2 Genetic Contributions of Three Additional Lineages Into the Lake Malawi Ancestral Hybrid Swarm

To test for hybridization events contributing genetic material to the Malawi cichlid radiation, we used Patterson's D (ABBA‐BABA) and the f_4_‐ratio statistic (Green et al. 2010; Patterson et al. 2012; Malinsky et al. 2021) to detect genome‐wide excess allele sharing in species trios ([P1, P2], P3), where P1 is a Victoria group species, P2 is a Malawi species and P3 is a non‐Malawi species. Trios with a significantly positive D represent an excess of shared derived alleles (likely explained by genetic exchange) between species P2 and P3, whereas significant negative D indicates excess allele sharing between species P1 and P3. We found that of these 321,216 tests, 50.3% (161,513 tests) had a significantly positive D statistic (Bonferroni FWER < 0.05), indicating significant Malawi/non‐Malawi excess allele sharing, whilst 39.0% (125,216 tests) had a significantly negative D statistic, indicating significant Victoria/non‐Malawi excess allele sharing.

**FIGURE 2:**
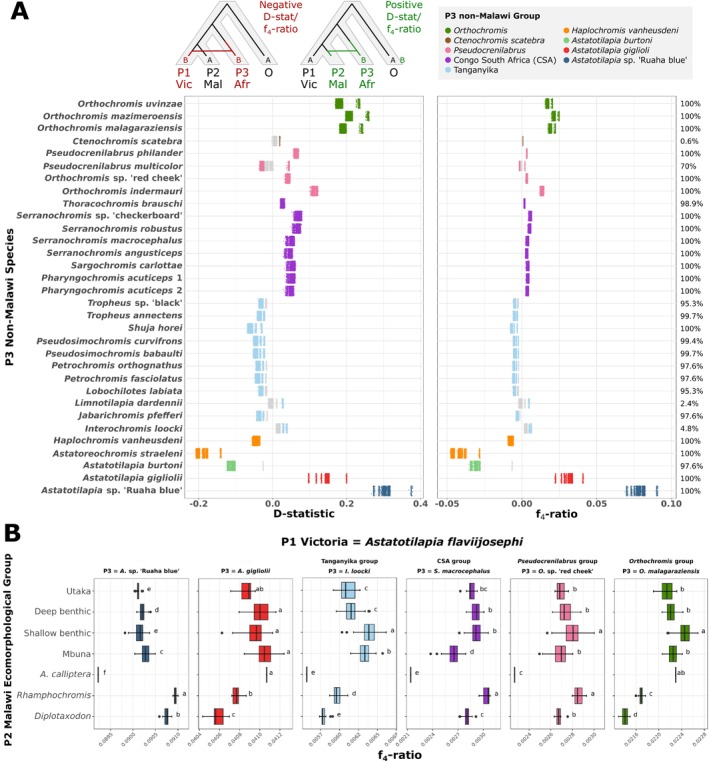
Potential genetic contributions of non‐Malawi African pseudocrenilabrine cichlids into the Malawi radiation. (A) ABBA‐BABA statistics (D‐statistic and f_4_‐ratio) for tests where species P1 = Victoria (VIC) and P2 = Malawi (MAL, positive values) or P1 = Malawi and P2 = Victoria (negative values), and P3 = non‐Malawi (AFR). Non‐significant test trios are marked in grey and significant trios are coloured according to the non‐Malawi group the P3 species is from. Significant positive tests indicate significant excess allele sharing between Malawi species and the P3 non‐Malawi species indicated, whereas significant negative tests indicate significant excess allele sharing between Victoria group species and the P3 species. On the right‐hand side is the percentage of trios that are significant for each P3 species. (B) f_4_‐ratios of significant trios where species P1 = *Astatotilapia flaviijosephi* (Victoria group), P2 = Malawi, and P3 = non‐Malawi species *A*. sp. ‘Ruaha blue’, *A. gigliolii*, *I. loocki* , *O*. sp. ‘Red cheek’ or *O. malagaraziensis* . The six chosen P3 non‐Malawi species were selected as representatives for their respective groups. Trios are separated into one of the seven Malawi ecomorphological groups (*Diplotaxodon*, *Rhamphochromis*, *A. calliptera* , ‘mbuna’, shallow benthic, deep benthic, and ‘utaka’). Boxplots are coloured according to the P3 non‐Malawi group. Pairwise significant differences in the mean f_4_‐ratio between groups (Tukey's HSD test, *p* < 0.05) are indicated using letters to the side of each box; groups with different letters have significantly different means, whilst groups that share a letter do not.

A single gene flow event can lead to several significant D statistics when related trios are considered. Therefore, we interpreted the results in the context of the monophyletic non‐Malawi species groups defined in Figure 1B. Four non‐Malawi groups at varying phylogenetic distances—*A*. sp. ‘Ruaha blue’, *A. gigliolii*, *Orthochromis* and CSA (Congo South Africa) – showed consistent excess allele sharing with the Malawi radiation. In other words, all possible combinations of Victoria P1 and Malawi P2 species were significant for all non‐Malawi P3 species in these groups (Figure 2A). Of these four groups, *A*. sp. ‘Ruaha blue’ showed the highest f_4_‐ratio, a statistic that estimates the proportion of the genome affected by introgression (mean = 8.0%, minimum = 6.9%, maximum = 9.1% across trios; Figure 2A). Variation in f_4_‐ratio (and D‐statistic) values among *A*. sp. ‘Ruaha blue’ trios depended on the chosen Victoria P1 species, with tests including Victoria species from the Rufiji sub‐basin (*A*. sp. ‘Ruaha red cheek’ and *Astatotilapia* sp. ‘longfin’) and the Lake Tanganyika affluent (*Haplochromis paludinosus*) yielding the lowest values and *Astatotilapia flaviijosephi* yielding the highest (Figure S5). This is consistent with secondary gene flow between co‐occurring members of the Victoria group and *A*. ‘Ruaha blue’, including the evidence of hybridization between *A*. ‘Ruaha red cheek’ (which is from the Victoria group), reported above.

The second largest set of f_4_‐ratios was associated with the *A. gigliolii* group (mean = 3.3%, minimum = 2.2% and maximum = 4.1%; Figure 2A). As above, the variation among trios in f_4_‐ratios depended on the Victoria P1 species, with approximately the same species showing the lowest and highest values (Figure S6). We interpret this pattern not as an additional gene flow event, but instead as a reflection of gene flow from *A*. sp. ‘Ruaha blue’, the *A. gigliolii* sister lineage, into the Lake Malawi radiation (though we present evidence for additional independent genetic exchange between *A. gigliolii* and specific Malawi lineages below).

Whilst the above signals of excess allele sharing with Malawi were reported previously by Svardal et al. (2020), here we identified two additional clear and consistent signals. Specifically, Malawi species showed strong excess allele sharing with (i) the *Orthochromis* group, which contains three species from the genus *Orthochromis* found in eastern Lake Tanganyika tributaries; and (ii) the CSA group, which contains several genera—including *Serranochromis* species from the Lake Malawi and Zambezi catchment. These two signals are likely independent as they come from distinct phylogenetic groups. The signals of gene flow between the Malawi radiation and *Orthochromis*, a group phylogenetically distant from Malawi, were particularly strong, with Patterson's *D* values of up to 26.2%, the second highest D statistics after those recorded for *A*. sp. ‘Ruaha blue’.

The f_4_‐ratios for *Orthochromis* trios were comparatively lower with a maximum of 2.5%. However, f_4_‐ratios have been shown to underestimate the amount of actual gene flow as the phylogenetic distance between the sister species P1/P2 and P3 increases (Martin et al. 2015; Svardal et al. 2020). Therefore, our results suggest the presence of at least 2.5% of genetic exchange between a lineage related to the *Orthochromis* group and an ancestor of Lake Malawi cichlids. The CSA group, with D statistic values up to 8.1% and f_4_‐ratio values up to 0.6% respectively, also showed highly significant excess allele sharing with Malawi, indicating a small but significant genetic exchange between a lineage related to the CSA group and the Malawi radiation.

Several other non‐Malawi groups also showed signals of excess allele sharing with Malawi that were statistically significant, but not consistent across species comparisons (Figure 2A). The most prominent was in the *Pseudocrenilabrus* group (not to be confused with the larger Pseudocrenilabrini group) containing specimens from several genera covering different river catchments around Lakes Victoria, Tanganyika and Malawi. All *Pseudocrenilabrus* species showed significant excess allele sharing signals with Malawi, indicating a gene flow event between a *Pseudocrenilabrus* ancestor and Malawi. However, we also observed a high variance in D statistic and f_4_‐ratio values across *Pseudocrenilabrus* species. Among those, the strongest signals were found for *Orthochromis indermauri* with a maximum D statistic of 0.12 and a maximum f_4_‐ratio of 1.5%. Note that despite its genus assignment, *O. indermauri* is not phylogenetically within the *Orthochromis* group. The second strongest signal was found for *Pseudocrenilabrus philander* (maximum D statistic = 0.071, maximum f_4_‐ratio = 0.36%), the most genetically distant *Pseudocrenilabrus* species to *O. indermauri*. The sister species to *P. philander* , *Pseudocrenilabrus multicolor* , showed only significantly positive D statistics in trios where P1 is the Victoria species *Astatotilapia flaviijosephi* , the most basal and geographically isolated species of the Victoria group. The more dominant signal for *P. multicolor* was that it showed mostly negative D statistics significant only for trios where P2 is a LVRS species (a subgroup of the Victoria group), likely indicating gene flow between the LVRS and *P. multicolor* (which occurs in the Lake Victoria region) or a related lineage. Overall, for the *Pseudocrenilabrus* group, we find signals of gene flow with both the Malawi radiation and the LVRS, the latter signal likely reducing positive D statistic and f_4_‐ratio values for all other *Pseudocrenilabrus* species. The effect of this would be the weakest for the *Pseudocrenilabrus* group member most distantly related to *P. multicolor* , as is observed with *O. indermauri*. We conclude that despite the magnitude and causing variation in excess allele sharing signals, there is an overall signal of the *Pseudocrenilabrus* lineage with the Malawi radiation.

Finally, for the Tanganyika group, which contains species endemic to Lake Tanganyika and its tributaries, there was an overall pattern of significant excess allele sharing affecting all Victoria group species (Figure 2A). However, two Tanganyikan species, *Limnotilapia dardennii* and *Interochromis loocki*, showed the opposite pattern, with significant excess allele sharing with Malawi and f_4_‐ratios of up to 0.6%. These significant positive tests were only for trios where the Victoria P1 species was *Astatotilapia flaviijosephi* or *Astatotilapia bloyeti* (both relatively basal in the Victoria group). This result is consistent with genetic exchange between *L. dardennii* and *I. loocki* with the Malawi radiation, but potentially overshadowed by gene flow events between the Tanganyika and Victoria groups. The fact that *L. dardennii* and *I. loocki* trios were significant only in comparisons with the relatively basal and geographically peripheral Victorian group species, that are not part of the Lake Victoria catchment ( *A. flaviijosephi* and *A. bloyeti*), indicates that Tanganyika/Victoria gene flow event occurred after the split of *A. bloyeti* from the remaining Victoria group.

Finally, *Ctenochromis scatebra* also showed weak excess allele sharing with Malawi species, but again only for trios with *A. flaviijosephi* as P1 and only for benthic Malawi species (i.e., from the ‘mbuna’, shallow benthic, deep benthic and ‘utaka’ groups) as P2.

### 3.3 Amounts of Genetic Exchange With Non‐Malawi Groups Are Similar Across the Radiation

Next, we estimated variability in signatures of genetic exchange between Lake Malawi species and other pseudocrenilabrine cichlid groups. We present results in the context of seven genetic groups of Malawi cichlids: *A. calliptera* , ‘mbuna’, shallow benthic, deep benthic and ‘utaka’—to which we will collectively refer as ‘benthics’—and the ‘pelagic’ groups *Diplotaxodon* and *Rhamphochromis* (Malinsky et al. 2018).

For some comparisons, the absolute strength of excess allele sharing as measured by the f_4_‐ratio differed significantly between Malawi groups (Tukey's HSD test) (Figure 2B, Figure S7). For example, for trios where P1 = *A. flaviijosephi* (Victoria group), the pelagic species groups *Diplotaxodon* and *Rhamphochromis* showed significantly higher f_4_‐ratios with P3 species *A*. sp. ‘Ruaha blue’ compared to all other Malawi groups, but significantly lower values with *A. gigliolii* and the *Orthochromis* and Tanganyika groups.

Furthermore, *A. calliptera* showed considerably lower f_4_‐ratios compared to the other Malawi groups in comparisons with several non‐Malawi species, including *A*. sp. ‘Ruaha blue’, *L. dardennii* and *I. loocki* from the Tanganyika group, all species from the CSA group (except *Thoracochromis brauschi* ), and *O. indermauri* and *Orthochromis* sp. ‘red cheek’ from the *Pseudocrenilabrus* group.

Importantly, however, differences among Malawi groups in the strength of excess allele sharing signals were relatively minor on an absolute scale. Therefore, we conclude that the majority of introgressed genetic material is shared across the Malawi radiation.

### 3.4 Recent Population Specific Gene Flow Between the Malawi Cichlid *Astatotilapia calliptera* and Riverine Astatotilapia Gigliolii

Next, we conducted a search for gene flow events which occurred after the onset of the Malawi radiation, and therefore affected specific Malawi lineages, using tests where P1 and P2 were both Malawi species, and P3 a non‐Malawi lineage as a potential gene flow donor. The results of these tests (Note S1) pointed towards the possibility that the Malawi species *A. calliptera* shares excess ancestry with the non‐Malawi riverine *A. gigliolii*. Despite being part of the Lake Malawi radiation, *A. calliptera* is broadly distributed in the Zambezi basin, and co‐occurs with *A. gigliolii* in the Rovuma river catchment, part of the East Central Coast region adjacent to Lake Malawi. To test for gene flow between the species in the Rovuma catchment, we split *A. calliptera* samples by population (19 populations) and considered the five *A. gigliolii* individuals separately for ABBA‐BABA tests. We found that all trios where P1 = *A. calliptera* non‐Rovuma or other Malawi species, P2 = *A. calliptera* Rovuma and P3 = *A. gigliolii* showed significant D statistics (mean = 0.39, maximum = 0.61, minimum = 0.21), with f_4_‐ratio values up to 2.7% (mean = 1.4%, minimum = 0.73%), consistent with gene flow between Rovuma *A. calliptera* and *A. gigliolii*.

**FIGURE 3:**
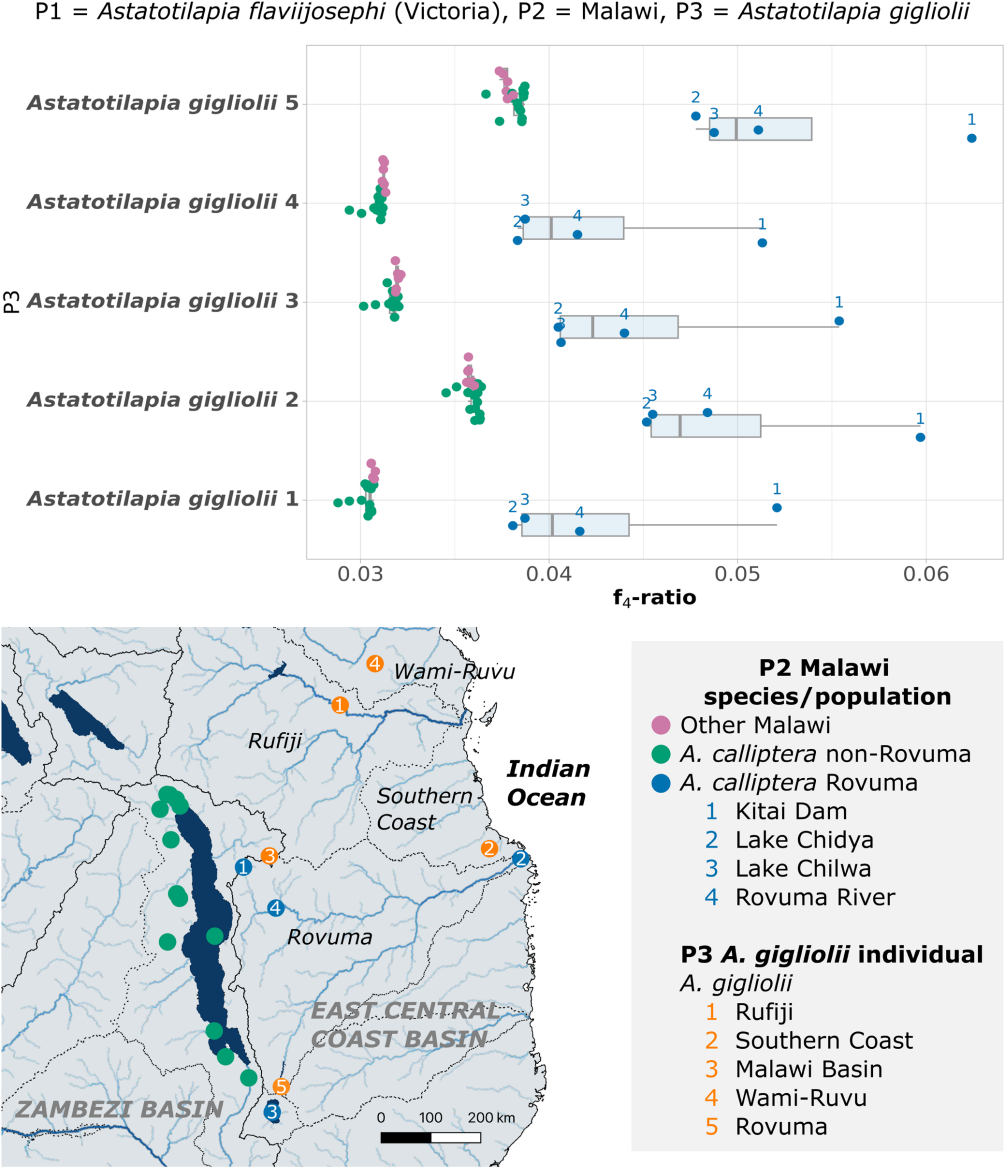
Recent gene flow between Rovuma *Astatotilapia calliptera* populations and *Astatotilapia gigliolii*. f_4_‐ratio of significant tests (100% of all tests) where P1 = *Astatotilapia flaviijosephi* (Victoria group), P2 = Malawi ( *L. fuelleborni* , *C. chrysonotus* , *D. limnothrissa* , *M. subocularis*, *A. peterdaviesi* and *R. woodi), Astatotilapia calliptera* populations from the Rovuma catchment or *Astatotilapia calliptera* populations not from the Rovuma, and P3 = *A. gigliolii*. Sampling locations of *A. calliptera* and *A. gigliolii* samples are shown below.

Given that gene flow between *A. calliptera* and *A. gigliolii* likely took place in the Rovuma catchment, we investigated whether gene flow was so recent that there would be variation in *A. gigliolii* introgression across Rovuma populations. We tested this with trios where P1 = *A. flaviijosephi* (Victoria), P2 = Malawi, non‐Rovuma or Rovuma and P3 = *A. gigliolii*. Consistent with recent population‐specific gene flow, we found considerable variation in f_4_‐ratio values among Rovuma populations, with the ‘Kitai Dam’ population showing consistently the highest f_4_‐ratio values (maximum = 6.2%, mean = 5.6%, minimum = 5.1%), followed by populations ‘Rovuma River’, ‘Lake Chilwa’ and then ‘Lake Chidya’ (Figure 3). Despite this variation, both a Principal Component Analysis (PCA) and ADMIXTURE analysis demonstrated little signs of recent hybridization between *A. calliptera* Rovuma samples with *A. gigliolii* (Note S2, Figure S8–S10), suggesting that the hybridization event is relatively old, but more recent than the split between *A. calliptera* Rovuma populations and non‐Rovuma populations.

### 3.5 Non‐Malawi Genetic Contributions Are Interspersed Across Malawi Cichlid Chromosomes

We asked how the signals of excess allele sharing are distributed along the genomes of Malawi cichlids. We calculated the statistic *f*_dM_ in non‐overlapping windows of 50 informative SNPs for trios where P1 contained all Victoria group species, P2 contained all Malawi species and P3 contained the groups with consistent signals of excess allele sharing with the Malawi radiation: *A*. sp. ‘Ruaha blue’, *A*. *gigliolii*, *Orthochromis*, CSA or *Pseudocrenilabrus*. When comparing all *f*_dM_ window values for a given trio, positive skews or shifts to positive values indicate signals of introgression between P3 (non‐Malawi) and P2 (Malawi), whilst negative skews or shifts would indicate signals of introgression between P3 and P1 (Victoria).

**FIGURE 4:**
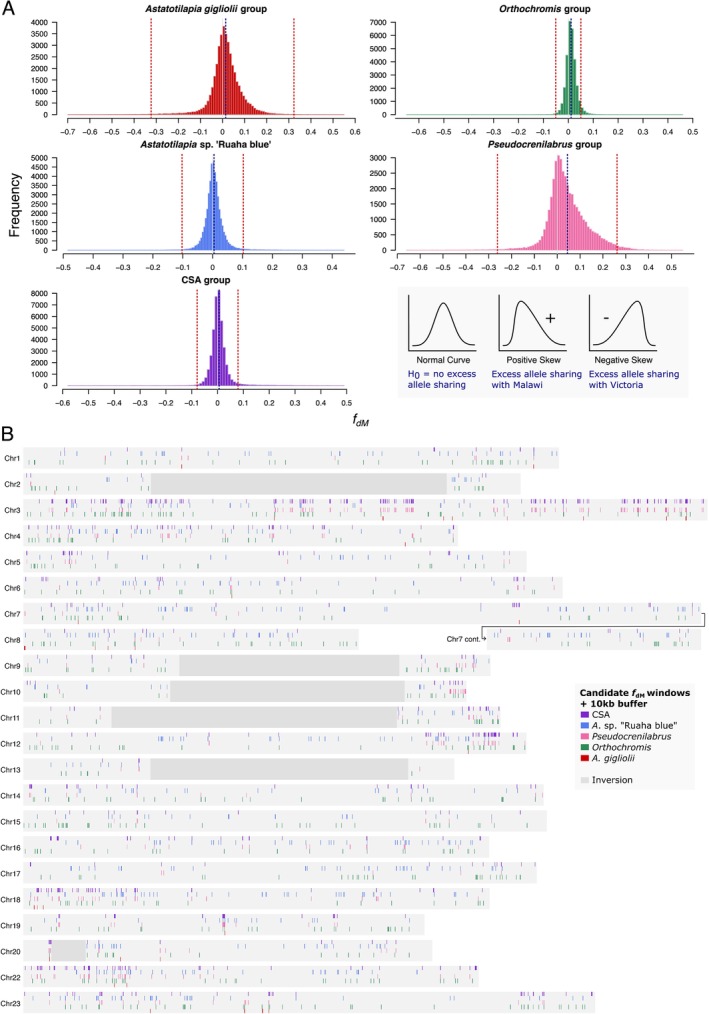
Signatures of gene flow in windows along the genome. (A) Dinvestigate *f*_dM_ windows of 50 snps for trios where species P1 = Victoria group, P2 = Malawi and P3 = *Astatotilapia gigliolii* group, *Orthochromis* group, *Astatotilapia*. sp. ‘Ruaha blue’, *Pseudocrenilabrus* group or CSA group. Windows with excess allele sharing between Malawi and a non‐Malawi group have a positive *f*_dM_ value, whereas windows with excess allele sharing between Victoria and a non‐Malawi group have a negative *f*_dM_ value. The blue dotted line marks the mean *f*_dM_ value across all windows for each P3 group and the red dotted line marks the threshold of significance (top and bottom 0.05%), used to select candidate windows for gene flow. (B) Distribution of candidate *f*_dM_ 50snp windows across the genome. For visualisation purposes, windows are artificially enlarged by a 10 kb buffer (adding 10 kb before the start and after the end of each window). Windows are coloured according to which P3 non‐Malawi group shows significant excess allele sharing with Malawi; CSA = purple, *A*. sp. ‘Ruaha blue’ = blue, *Pseudocrenilabrus* = pink, *Orthochromis* = green and *A. gigliolii* = red.

As expected, we found a significant deviation from a normal distribution with a positive skew for all P3 groups tested (*A. gigliolii* D_KS_ = 0.107, *p* < 0.001; *Orthochromis* D_KS_ = 0.674, *p* < 0.001; *A*. sp. ‘Ruaha blue’ D_KS_ = 0.108, *p* < 0.001; *Pseudocrenilabrus* D_KS_ = 0.0748, *p* < 0.001; CSA D_KS_ = 0.15331, *p* < 0.001; one‐sample Kolmogorov–Smirnov tests), indicating that signals of excess allele sharing between the Malawi radiation and non‐Malawi species are detectable also by this local statistic in windows along the genome (Figure 4A). Of all groups tested, the *Pseudocrenilabrus* group showed the strongest shift in mean *f*_dM_ value (*f*_dM_ means per group: *A. gigliolii*, 0.015; *Orthochromis*, 0.011; *A*. sp. ‘Ruaha blue’, 0.003; *Pseudocrenilabrus*, 0.045; CSA, 0.003), indicating the strongest signal of gene flow at the window specific level across all groups.

The main use of the *f*_dM_ statistic is to identify candidate introgressed regions in cases where the genome‐wide D statistic is significantly positive. However, given that variation in local *f*_dM_ values arises through a combination of incomplete lineage sorting, sampling noise and introgression, it is difficult to choose a biologically meaningful cutoff. To select candidate introgressed windows, we used an approach based on the difference between the distributions of positive and negative *f*_dM_ values (Figure 4A). The number of candidate introgressed windows varied greatly among the groups: from 49 for *A. gigliolii* to 980 for the CSA group, while the percentage of the genome encompassed by these windows varied from 0.16% to 4.75% (Table 1).

**TABLE 1:** Candidate windows for gene flow between Malawi and P3 non‐Malawi groups.

P3 group	Total windows	Average window size (kb)^a^	Average candidate window size (kb)^b^	Candidate windows (%)^c^	No. candidate windows	No. adjacent candidate windows^d^	No. expected adjacent candidate windows^e^	Deviation from expected adjacent windows^f^	Adjacent candidate windows (%)	No. of candidate regions of adjacent window length *n* where *n* is:^g^												
										2	3	4	5	6	7	8	9	10	11	12	13	14
*A. gigliolii*	30,010	12.95	8.63	0.16	49	21	0.16	131.24	43	5	2	0	1	0	0	0	0	0	0	0	0	0
*Orthochromis*	14,020	15.80	13.83	4.75	666	97	63.27	1.53	15	42	3	1	0	0	0	0	0	0	0	0	0	0
*Pseudocrenilabrus*	39,861	12.63	7.74	1.33	531	227	14.15	16.05	43	52	23	5	4	1	0	1	0	0	0	0	0	0
*A*. sp. ‘Ruaha blue’	34,477	13.09	10.45	2.01	693	138	27.86	4.95	20	52	10	1	0	0	0	0	0	0	0	0	0	0
CSA	31,441	13.80	8.84	3.12	980	611	61.09	10.00	62	107	44	18	12	1	5	1	4	1	0	2	0	1

^a^ Average window size of all non‐overlapping 50 SNP windows in the Dinvestigate analysis. Window size varies depending on the density of snps.

^b^ Windows were marked as candidates for gene flow between Malawi and the P3 group if they had an *f*_dM_ value higher than the absolute *f*_dM_ value which marks the boundary for the most negative 0.5% of windows.

^c^ Percent of candidate windows out of all windows for a P3 group.

^d^ Candidate windows were marked as adjacent when positioned in the genome directly before or after one or more other candidate windows for the same P3 group.

^e^ Expected number of candidate windows which are adjacent to one or more other candidate windows (Total no. of windows −1 × (No. candidate windows/Total no. of windows)^2^ × 2).

^f^ Deviation of the observed number of adjacent candidate windows from the expected number (expected number of adjacent candidate windows/observed number of adjacent candidate windows).

^g^ Candidate regions are uninterrupted stretches of adjacent candidate windows of length *n* windows. Candidate regions are counted, not the number of windows within a candidate region, that is, 1 region of length 8 candidate windows will have a count of 1.

Whilst candidate windows appeared visually to be widely distributed across the genome, without any clear clustering in outlier regions (Figure 4B, Figure S11), statistical examination revealed that, for all P3 groups, there were significantly more candidate windows that were adjacent to each other than expected by chance (Table 1). The pattern was particularly striking for the CSA group, with 62% of all candidate windows occurring in blocks of up to 14 adjacent candidate windows. *Pseudocrenilabrus* and *A. gigliolii* also showed a strong enrichment, with both showing 43% adjacent candidate windows, in blocks of up to eight adjacent windows. Given average window sizes of ~10 kb, this suggests the presence of relatively long haplotypes (up to ~140 kb) that show signals of genetic exchange with non‐Malawi lineages.

## 4 Discussion

With the increasing availability of genomic data, we now know that genetic exchange between diverging lineages is common across the animal and plant kingdoms, especially in adaptive radiations (Martin et al. 2013; Lamichhaney et al. 2015; Mallet et al. 2016; Novikova et al. 2016; Svardal et al. 2017). The prevalence of introgression in adaptive radiations could be a consequence of the fact that the radiations consist of many closely related species found often in a confined area, creating many opportunities for hybridization. However, a range of theoretical considerations and empirical findings, including from each of the major African cichlid radiations, show that gene flow can also promote speciation and diversification (Seehausen 2004; Consortium 2012; Schumer et al. 2014; Meier et al. 2017; Stryjewski and Sorenson 2017; Marques et al. 2019; Meier et al. 2019; Selz and Seehausen 2019; Svardal et al. 2020). This has contributed to a view that speciation, in adaptive radiations in particular, might often involve combinatorial shuffling of various pre‐adapted haplotypes facilitated by gene flow (Hedrick 2013; Marques et al. 2019).

In East African cichlids, individual instances of gene flow occurring close to the origins of their radiations have been interpreted as facilitating or ‘fuelling’ their onset (Meier et al. 2017; Irisarri et al. 2018; Marques et al. 2019; Svardal et al. 2020; Ronco et al. 2021). Furthermore, a recent study based on comprehensive geographic and taxonomic sampling has shown that the history of the Lake Victoria Region Superflock (LVRS) is characterised by multiple cycles of fusion and fission of lineages, generating several radiations (Meier et al. 2023). Here, we used a similarly comprehensive sample set to conduct a thorough search for contributions to the Lake Malawi cichlid radiation from other African pseudocrenilabrine cichlid lineages. We demonstrate that, alongside the known genetic contributions from a lineage related to the Victoria group (a clade containing the LVRS and several riverine pseudocrenilabrine species) and another one related to *Astatotilapia*. sp. ‘Ruaha blue’ (Svardal et al. 2020), at least three additional lineages—*Orthochromis*, Congo South Africa (CSA) and *Pseudocrenilabrus*—show consistent genome‐wide and window‐specific patterns of excess allele sharing with Malawi. We interpret our results as indicators of three independent gene flow events occurring between ancestors of these lineages and the common ancestor of the Lake Malawi radiation.

It is important to note that the precise gene flow events which produced the observed ABBA‐BABA results can be hard to fully disentangle when considering many closely related lineages and several gene flow events. For example, whilst we observed consistent significant excess allele sharing between the riverine *Astatotilapia gigliolii* and Malawi, these patterns closely mirrored those of *A*. sp. ‘Ruaha blue’. Therefore, like Svardal et al. (2020), we interpret the bulk of this result not as an additional gene flow event, but instead as a reflection of the gene flow patterns from the *A. gigliolii* sister lineage, *A*. sp. ‘Ruaha blue’. Also, whilst not a focus of this study, our analyses simultaneously detected interesting signals of gene flow between the Victoria group lineage and other pseudocrenilabrine groups tested, including Lake Tanganyika Tropheina, the riverine species *Pseudocrenilabrus multicolor, and A*. sp. ‘Ruaha blue’. Such gene flow events with Victoria have likely acted to tip the balance of ABBA/BABA sites in other related non‐Malawi species and artificially diminish the signatures of gene flow from other pseudocrenilabrine lineages with Malawi. This effect is clearly observed for *A*. sp. ‘Ruaha blue’ trios, where a high variance in D statistics is likely caused by recent gene flow between a Victoria species (likely *Astatotilapia* sp. ‘Ruaha red cheek’) and two *A*. sp. ‘Ruaha blue’ samples—which presently co‐occur in the Ruaha catchment within the Rufiji sub‐basin. Notably, despite this confounding effect, we continued to observe highly significant trios, indicating that the inferred gene flow events are noteworthy despite low D statistic values in some cases. The best example of this is in *Pseudocrenilabrus multicolor* , which simultaneously showed signals of gene flow with both the Malawi radiation and the LVRS. This then likely caused variance in and reduced the positive D statistic and f_4_‐ratio values for all other *Pseudocrenilabrus* species. Overall, we conclude that our results point towards many old and new gene flow events among pseudocrenilabrine lineages which in turn has resulted in an artificially diminished and complex pattern of excess allele sharing. Despite this, we observed clear and consistent signals of gene flow with Malawi in three groups, the *Orthochromis*, *Pseudocrenilabrus* and CSA groups, which we focus on in our discussion.

An important question is the directionality of gene flow of non‐Malawi groups with Malawi—specifically, whether outgroups genetically contributed to the ancestral Malawi radiation, whether a Malawi lineage genetically contributed to outgroups, or whether gene flow occurred in both directions. Unfortunately, our phylogenetic setup does not allow for the use of the five‐taxon test (Pease and Hahn 2015), an established statistic to test for the directionality of gene flow. Other methods, like model‐based inference, could potentially shed light on this question, but given the complexity of our study system, with many speciation events and complex demographic histories separating extant lineages and frequent gene flow connecting them, their application is not straight forward and will need to be reserved for future more focused studies.

That said, we think it is likely that one or more of the reported gene flow events went in the direction of the Malawi ancestor and thus contributed to the previously reported ancestral hybrid swarm. In ABBA‐BABA tests, signals of gene flow can only be detected in direct descendants of the recipient lineage, but it is not necessary to sample the actual donor lineage—a related lineage can act as a surrogate. The groups showing consistent evidence for gene flow with Malawi—*Orthochromis*, *Pseudocrenilabrus* and CSA—all have wide geographic distributions while the Malawi lineage is currently chiefly limited to the Lake Malawi basin. Although the geological history of East Africa is dynamic and our knowledge of historic distributions and dispersal patterns of pseudocrenilabrine lineages is limited, it seems more parsimonious that local relatives of geographically widespread groups (*Pseudocrenilabrus* and CSA species occur in the Malawi basin) contributed to the Malawi radiation than that an ancestor or relative of the Malawi radiation contributed to each member of these widespread groups or their direct ancestors.

More specifically, for the case of the *Pseudocrenilabrus* group, we estimated the split time to be substantially older than that of the Malawi/Victoria split (6.99–7.06 Mya vs. 4.56–4.65 Mya, node D vs. node B in Figure 1B), meaning that it is unlikely that all *Pseudocrenilabrus* species are direct descendants of a recipient lineage. This scenario is supported by findings from Blumer et al. (2025) who, using a similar callset to this study with the same non‐Malawi specimens, showed that an adaptive genomic contribution from a lineage related to *P. philander* into Malawi benthic species was retained in a genomic inversion.

The gene flow events described here involve lineages which diverged millions of years ago. By comparison, the gene flow events that contributed to the origins of the LVRS, as discussed by Meier et al. (2017) and Meier et al. (2023) are among lineages within our ‘Victoria group’ and thus substantially more recent. Comparing average sequence divergence between these lineages, we estimate the CSA, *Pseudocrenilabrus* and *Orthochromis* lineages to be respectively 3.1, 3.4 and 2.8 times more divergent from Malawi than the Upper Nile lineage is from the LVRS (*A*. sp. ‘Ruaha blue’ is 2.2 times more divergent) and 4.6, 5.0, 4.2 times more divergent than the Congolese lineage is to the LVRS (*A*. sp. ‘Ruaha blue’ is 3.3 times more divergent). While it is important to keep in mind that gene flow happened in historic times between ancestral lineages, this finding suggests that pseudocrenilabrines retained their ability to hybridise over larger phylogenetic distances than previously thought. Previous studies have proposed an ‘Out of Tanganyika’ origin for pseudocrenilabrine cichlids, suggesting that the tribe originally diversified in Lake Tanganyika (Salzburger et al. 2005) or—before its formation—in an area east of it (Schedel et al. 2019; Altner et al. 2020) and subsequently spread over different parts of eastern and southern Africa. Given this phylogeographic evidence, it is plausible that the broader Lake Tanganyika region has been point of contact between the ancestral Malawi lineage, and its new riverine genetic donors.

Importantly, we note that the amount of genetic material shared in these gene flow events is likely to be significantly underestimated in our analyses. Svardal et al. (2020) showed that the f_4_‐ratio of 10% (maximum 9.1% found in this study) between Malawi and *A*. sp. ‘Ruaha blue’ corresponds to 22% actual gene flow, based on coalescent simulations and genome‐wide proportions of Ruaha‐blue‐like haplotypes. The underestimation of gene flow proportions by the f_4_‐ratio also increases with increasing phylogenetic distance between species P1/P2 and P3 (Martin et al. 2015; Svardal et al. 2020). Therefore, we expect that the modest f_4_‐ratios calculated for the *Orthochromis*/CSA/*Pseudocrenilabrus* lineages, at between 0.1% and 2.5%, underestimate actual gene flow proportions even more so than for *A*. sp. ‘Ruaha blue’, and as such could likely represent significant genomic contributions to the Malawi ancestral lineage.

In addition to the ancient radiation‐wide signals described above, we observed two noteworthy cases of more recent, lineage‐specific gene flow in riverine species. First, outside of the Malawi radiation, we found signals of contemporary gene flow between two *A*. sp. ‘Ruaha blue’ samples with a Victoria ‘*Astatotilapia*’ riverine lineage, likely *A*. sp. ‘Ruaha red cheek’, in the Rufiji River basin that is part of the East‐Central Coast Region. Secondly, involving the Malawi radiation, we discovered recent genetic exchange between *A. gigliolii* and the Malawi species, *Astatotilapia calliptera*, which inhabits shallow macrophyte‐rich littoral habitats in Malawi and adjacent satellite lakes and rivers. This gene flow signal was observed only in *A. calliptera* populations that occur in the rivers of the East‐Central Coast. These findings are significant because of the ‘transporter hypothesis’ posited by Loh et al. (2013), who, based on rampant signals of shared polymorphisms across pseudocrenilabrines among 280 SNP loci, suggested that riverine cichlid species, in particular *Astatotilapia* riverines, could be important transporters of genetic polymorphism between lake radiations. Until now, however, no clear cases of contemporary riverine hybrids have been found. Our findings raise the possibility that haplotypes which evolved within the Lake Malawi radiation could be transported beyond its catchment area. Such transportation could potentially also go the other way, with *A. calliptera* acting to bring more recent non‐Malawi genetic variation into the radiation. Malinsky et al. (2018) showed that there have been frequent gene flow events within and between Malawi ecomorphological groups, including between *A. calliptera* populations from Lake Kingiri and the shallow benthic species *Otopharynx tetrastigma* in Lake Ilamba. Overall, these findings could support a potential role of ‘transporter’ riverine species in providing contemporary genetic variation to radiations.

To conclude, our study has revealed that the history of the Lake Malawi cichlid radiation is considerably more complex than previously appreciated, identifying multiple distinct and highly divergent pseudocrenilabrine lineages as potential genetic contributors to the Malawi ancestor, as well as evidence of more recent genetic exchange between a Lake Malawi lineage and a riverine relative. These findings highlight the importance of including a wide phylogenetic breadth of species in African cichlid genomic research. Future work should establish whether introgressed sequences have been under selection and contributed to adaptive phenotypes during the Lake Malawi radiation. We expect that the identification of source lineages and candidate introgressed regions will provide a firm basis for understanding how external gene flow contributed to adaptation and diversification in one of the largest vertebrate radiations.

## Author Contributions

S.G., M.M. and H.S. conceived the study. H.S., B.R., M.N., G.F.T. and M.J.G. collected samples. B.R. and M.N. facilitated acquiring permits. S.G. performed the analyses with input from M.M. and H.S. G.F.T. and M.J.G. contributed taxonomic expertise and results interpretation. S.G., H.S. and M.M. wrote the manuscript with comments from G.F.T. and M.J.G. All authors read and approved the manuscript.

## Disclosure

Benefit‐sharing statement: Genetic material and sequences are subject to Access and Benefits Sharing (ABS) agreement CS19014 with the Government of Malawi.

## Conflicts of Interest

The authors declare no conflicts of interest.

## Supporting information


Table S1.

Appendix S1.

## Data Availability

Raw sequencing reads are available on the SRA (see Table S1 for BioProject and BioSample numbers). Scripts for sequence alignment/variant calling/filtering, ML and NJ tree calculations and plotting, lineage split time calculations and Dtrios and Dinvestigate calculations and plotting are available at https://github.com/sophiegresham/MalawiGeneFlow. Genetic material and sequences are subject to an Access and Benefits Sharing (ABS) agreement with the Government of Malawi. Any person who wishes to use this data for any form of commercial purpose must first enter into a commercial licensing and benefit‐sharing arrangement with the Government of Malawi. The variant calling format (VCF) file EVA accession number, ML and NJ trees in Newick format, as well as the Dtrios output (BBAA.txt file) are available on zenodo (https://doi.org/10.5281/zenodo.15586284).
