# Supplementary material for: Genome Analyses Reveal Diverse Riverine Genetic Contributions to the Lake Malawi Cichlid Radiation
Source: Mol Ecol. 2025 Jun 10;34(13):e17786. doi: 10.1111/mec.17786 (PMC12186717; doi:10.1111/mec.17786)
Supplement: Supplementary file 2 — Appendix S1. [file MEC-34-e17786-s002.pdf]

MOLECULAR ECOLOGY

Supplemental Information for:

Genome analyses reveal diverse riverine genetic contributions to the Lake Malawi cichlid radiation

Sophie Gresham, Bosco Rusuwa, Maxon Ngochera, George Turner,
Martin Genner, Milan Malinsky, Hannes Svardal

Table of Contents:

**Title Page**	Page 1
**Supplementary Notes**	Pages 2-4
**Supplementary Figures**	Page 5-21

MOLECULAR ECOLOGY

Supplementary Notes

Supplementary Note 1

We conducted a search for differences between Malawi species/lineages in the excess allele sharing of non-Malawi ancestry, which could either indicate additional gene flow events that occurred more recently than the onset of the Malawi radiation and therefore affect only specific Malawi species/lineages, or could indicate differential retention of introgressed haplotypes due to selection (adaptive introgression). To do this, we computed genome-wide ABBA BABA statistics for all possible combinations of species where P1 and P2 were both Malawi species and P3 is a non-Malawi species. Any significant trios would indicate cases where one Malawi species (P2) has a significant excess allele sharing with a non-Malawi species (P3) relative to another Malawi species (P1), thus indicating gene flow events which affect one Malawi species but not another. Of the 910,112 trios which matched this criteria, 2.7% (24,394 tests) had a significantly positive D statistic (Bonferroni FWER < 0.05) with the maximum f_4-ratio across all significant tests at 0.3%, indicating low overall differential excess allele sharing signals across Malawi species.

To simplify analysis of comparisons, we first categorized combinations of Malawi P1 and P2 according to whether the P1 and P2 species were pelagic (from the *Diplotaxodon* or *Rhamphochromis* ecomorphological groups) or benthic (from the *A. calliptera*, 'mbuna', shallow benthic, deep benthic, or 'utaka' ecomorphological groups). We tested for differences within the pelagics and benthics (pelagic P1 vs pelagic P2 and benthic P1 vs benthic P2) and also between pelagics and benthics (pelagic P1 vs benthic P2 and benthic P1 vs pelagic P2). We found that the differences between Malawi species/ecomorphological groups in non-Malawi ancestry generally mirrored closely the differences found from the previous ABBA BABA tests where P1 = Victoria and P2 = Malawi. For example, for trios P1 = Victoria, P2 = Malawi, and P3 = *Orthochromis* group, we found that all benthic species showed significantly higher f_4-ratios than pelagic species, indicating higher excess allele sharing between the *Orthochromis* group and Malawi benthics (Supplementary Fig. 7). For trios where P1 = benthic and P2 = pelagic (and vice versa) with P3 = *Orthochromis* group, we found significant trios for trios where P1 = pelagic and P2 = benthic, meaning that some Malawi benthics have significant excess allele sharing with *Orthochromis* group species compared to pelagics (Supplementary Fig. 12, Supplementary Fig. 13). In another example, we found that for trios P1 = Victoria, P2 = Malawi, and P3 = *Astatotilapia* sp. "Ruaha blue", the pelagics species showed significantly higher f_4-ratios than the benthics, in particular for the benthic species *Astatotilapia calliptera*. This result was mirrored in trios where P1 = benthic and P2 = pelagic (and vice versa) with P3 = *Astatotilapia* sp. "Ruaha blue"; we found significant trios only in trios where P1 = benthic and P2 = pelagic, and all P1 = *A. calliptera* trios were significant, overall indicating that the Malawi pelagics have

significant excess allele sharing compared to benthics, which is particularly strong for the benthic *A. calliptera*.

Testing for differences within the pelagics and benthics also revealed differences that match those found in the P1 = Victoria/P2 = Malawi tests (Supplementary Fig. 14, Supplementary Fig. 15). However, we also found a striking enrichment of tests where P1 is *A. calliptera*, P2 is another benthic species (from the ecomorphological groups deep and shallow benthic, 'utaka', or 'mbuna') and P3 is almost any species from all non-Malawi groups (with exception of *Astatotilapia gigliolii* and two species from the *Pseudocrenilabrus* group) (Supplementary Fig. 15). Out of 15,423 significant tests for which P1 and P2 were benthic Malawi cichlids, 89% (13,760 tests) were with *A. calliptera* as the P1 species. These significant trios made up 42% of all significant trios across Malawi clades. This result indicates that almost all non-Malawi groups tested, except *A. gigliolii,* showed significant excess allele sharing with Malawi benthic species (including mbuna) relative to *A. calliptera*. However, since there is no common ancestor between the non-Malawi groups that does not also include Malawi, any gene flow events between these P3 groups and benthics would have to be independent of each other. A more likely explanation for this pattern is that *A. calliptera* has some other ancestry that it does not share with other benthic species. Two pieces of evidence suggest that it is the riverine *A. gigliolii* that has provided this ancestry; (1) *A. gigliolii* is the only P3 group which doesn't have an enrichment of *A. calliptera* P1 significant trios and (2) the strength of the enrichment (i.e. the percentage of significant trios where *A. calliptera* is P1 for benthic vs benthic tests) for each P3 group decreases with increasing genetic distance from *A*. sp. "Ruaha blue", the sister group to *A. gigliolii*.

Supplementary Note 2

To assess whether the observed excess allele sharing between *Astatotilapia gigliolii* and *Astatotilapia calliptera* populations from the Rovuma catchment indicates a very recent case of hybridisation and subsequent introgression, we performed a principal component analysis (PCA) and ADMIXTURE analysis on samples from these populations. The PCA analysis showed clear species differentiation between *A. gigliolii* and *A. calliptera* on PC1 (PVE = 35.7%) with no clear intermediates indicating recent hybrids (with a high proportion of both *A. gigliolii* and *A. calliptera* ancestry, Supplementary Fig. 9). However, on PC1, *A. calliptera* Rovuma samples did show values considerably closer to *A. gigliolii* than non-Rovuma populations did to *A. calliptera*, potentially indicating a weak signal of introgression. PC2 (PVE = 5.7%) showed a strong spread of *A. calliptera* samples indicating strong population differentiation, with the Rovuma populations showing clear differentiation from the non-Rovuma populations. Such a spread is typical for admixed populations. In other PC axis comparisons, we observed in some cases the clustering of *A. calliptera* Rovuma samples with *A. gigliolii* samples, but these clusters were not fully distinct from *A. calliptera* non-Rovuma samples. The ADMIXTURE analysis showed an optimal K model of 2 (Supplementary Fig. 10). In this K model, we observed a clear distinction

MOLECULAR ECOLOGY

between the two species; all *A. gigliolii* and *A. calliptera* samples showed ancestry proportions of 99.99% for their respective two ancestries. In higher K models there were no signatures of introgression in *A. calliptera* Rovuma samples. Overall, these analyses do not support very recent *A. gigliolii* introgression in the *A. calliptera* Rovuma populations, but do still support a weak signal of introgression specific to *A. calliptera* Rovuma. This result is harmonious with the small but significant f_4-ratios (mean = 1.4%) in ABBA BABA trios where P1 = *A. calliptera* non-Rovuma, P2 = *A. calliptera* Rovuma, and P3 = *A. gigliolii* (see main text).

MOLECULAR ECOLOGY

Supplementary Figures

MOLECULAR ECOLOGY

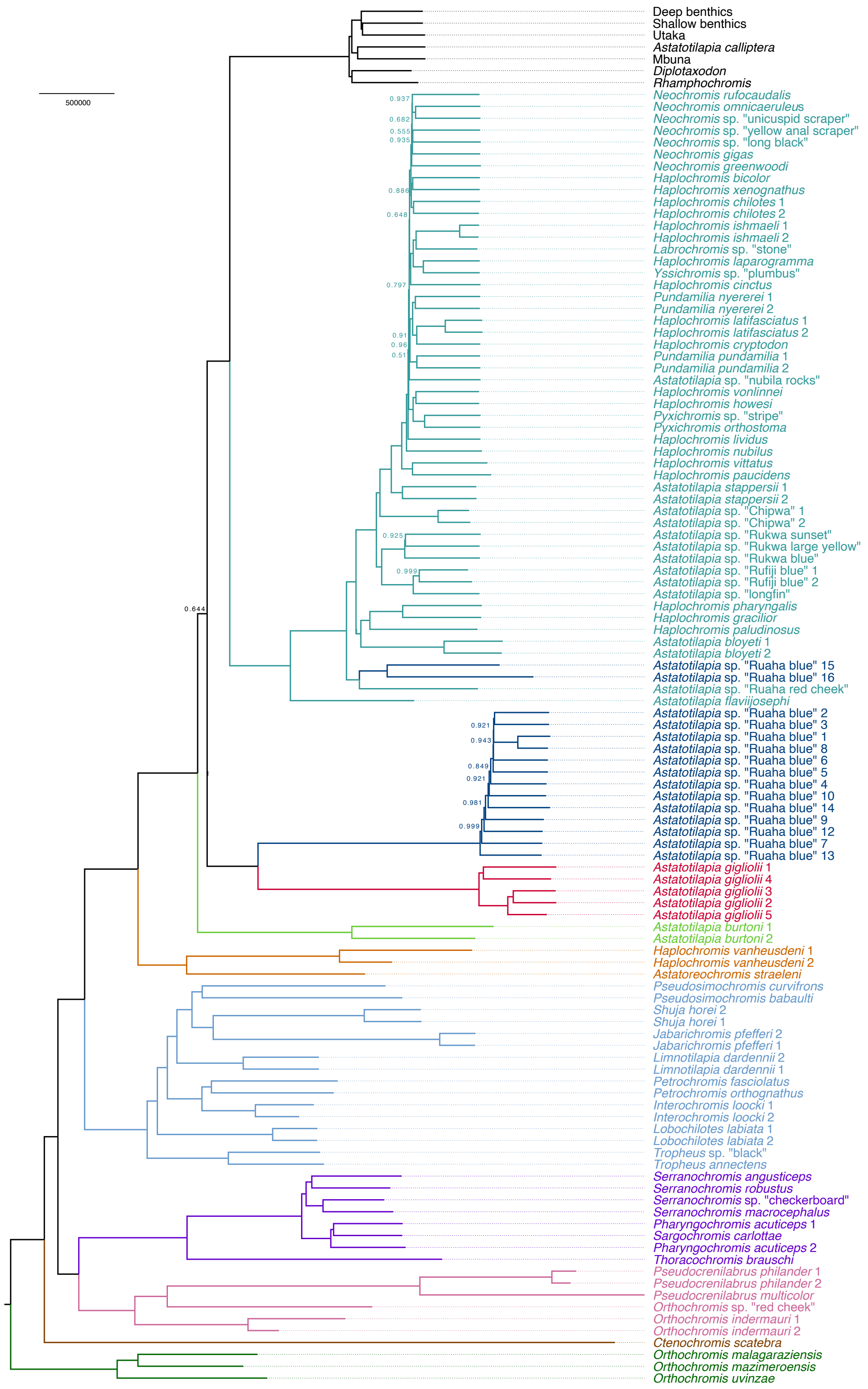

MOLECULAR ECOLOGY

Supplementary Figure 1. Consensus genome-wide neighbour joining phylogenetic tree calculated based on pairwise genetic differences for 612 specimens of Malawi and non-Malawi African cichlids in 100kb windows. The tree is rooted to a reconstructed ancestral state sample. Tip labels contain the species name, specimen number for that species (no number indicates only one specimen in the callset for the species). The Malawi radiation is collapsed into seven major clades. The scale bar gives total number of pairwise differences. Node values give the proportion of replicates which recovered the node in a bootstrapping analysis and range between 0 and 1, where 1 represents maximal support. Only nodes with a value lower than 1 are labelled.

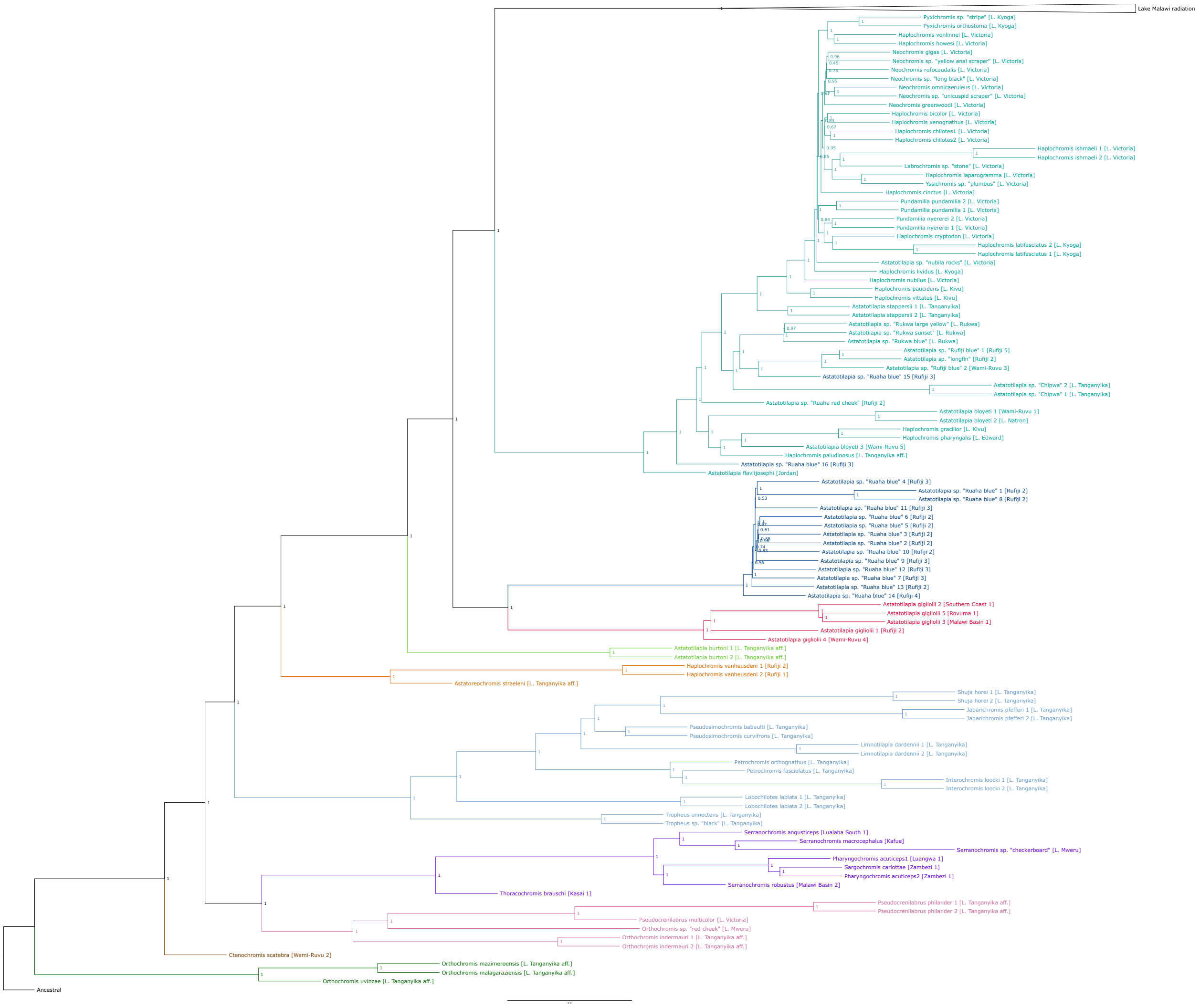

Supplementary Figure 2. Maximum likelihood phylogenetic tree built from whole-genome short-read sequences from 612 samples across 312 Malawi and Non-Malawi cichlid species, rooted to a reconstructed ancestral state sample. Tip labels contain the species name, specimen number for that species (no number indicates only one specimen in the callset for the species) and the general sampling location (in square brackets). All Malawi species are collapsed into a single clade. The scale bar gives genomic distance in coalescent units, and is based on the amount of discordance in gene trees. Nodes give the ASTRAL local posterior probability (LPP) value, which ranges between 0 and 1, where 1 represents maximal support.

MOLECULAR ECOLOGY

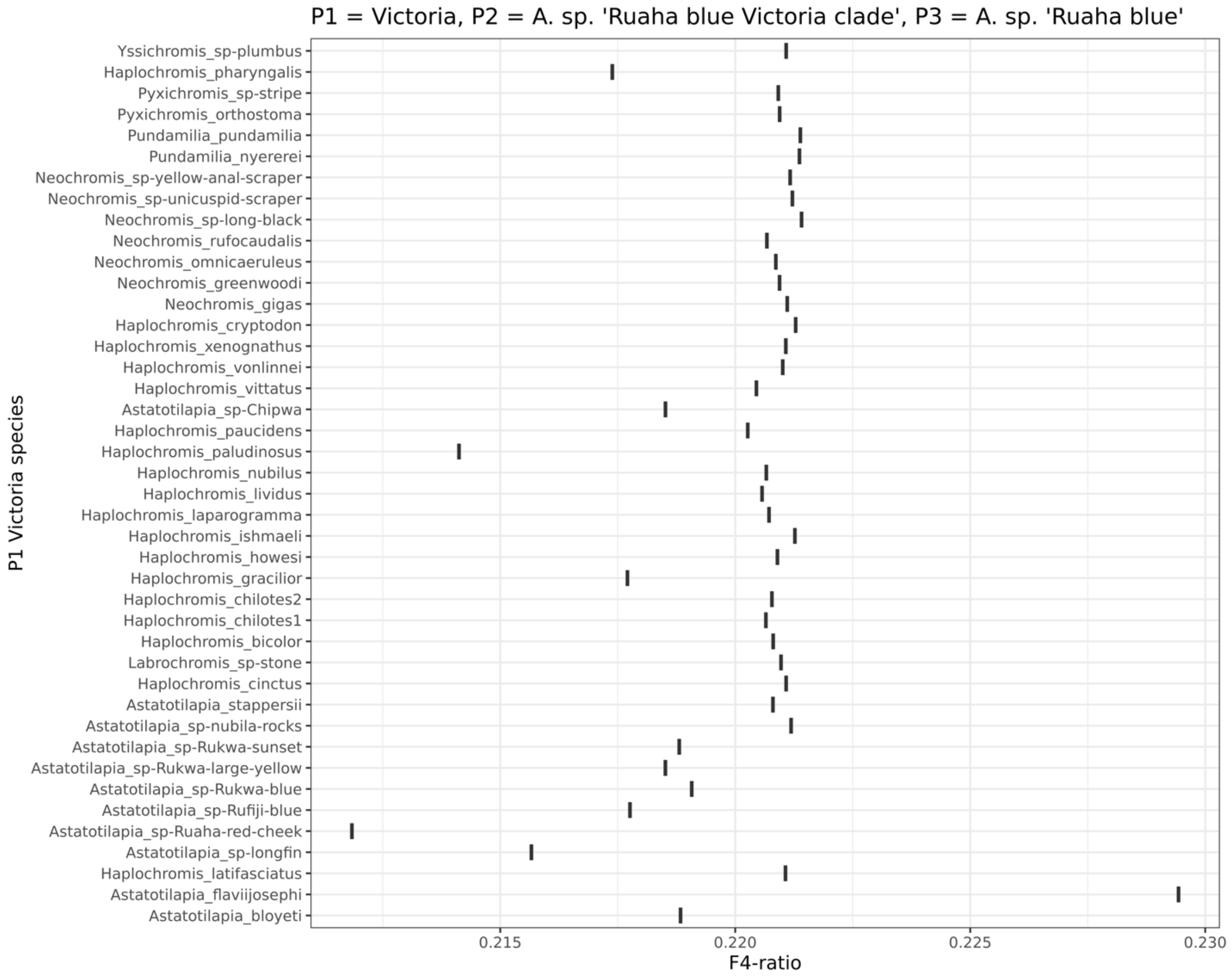

Supplementary Figure 3. F4-ratios of ABBA BABA tests where P1 = Victoria clade species, P2 = *Astatotilapia* sp. "Ruaha blue Victoria clade" (2 specimens that clustered within the Victoria clade in phylogenetic trees), P3 = *Astatotilapia* sp. "Ruaha blue" (14 specimens that clustered outside the Victoria clade). Only tests with a significant D-statistic are shown (100% of all 42 trios which match the species filtering criteria)

MOLECULAR ECOLOGY

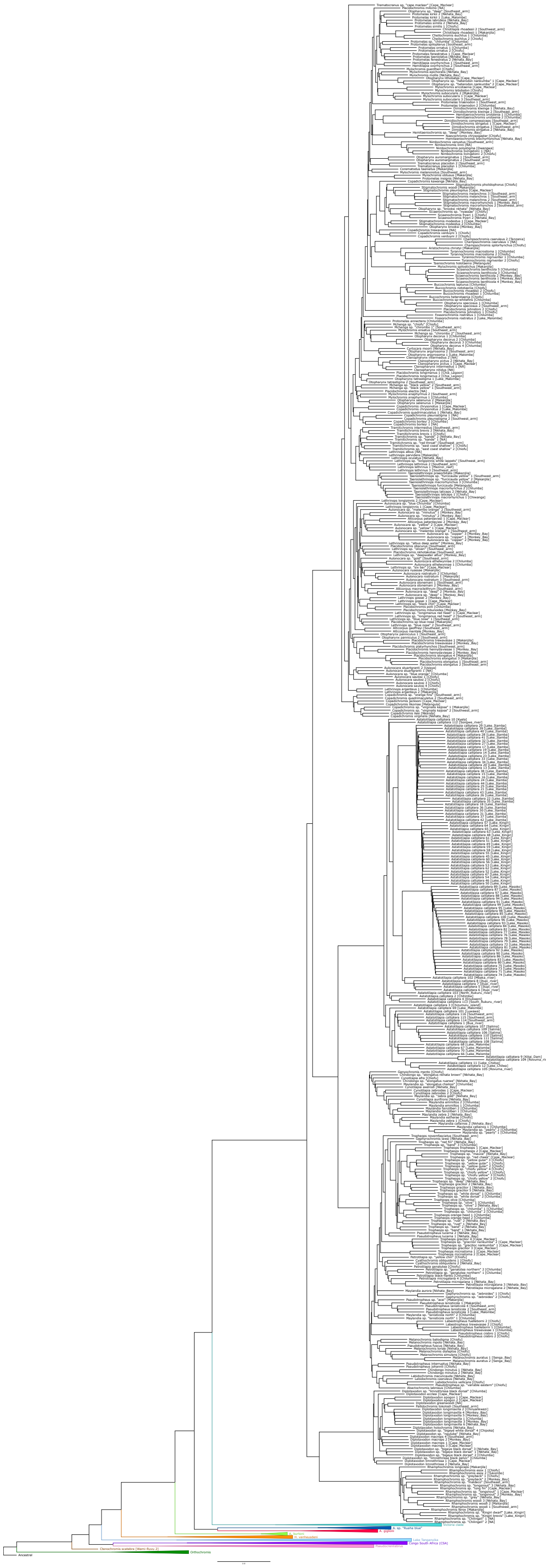

MOLECULAR ECOLOGY

Supplementary Figure 4. Maximum likelihood phylogenetic tree built from whole-genome short-read sequences from 612 samples across 312 Malawi and Non-Malawi cichlid species, rooted to a reconstructed ancestral state sample. Tip labels contain the species name, specimen number for that species (no number indicates only one specimen in the callset for the species) and the general sampling location (in square brackets). Non-Malawi species are collapsed coloured according to clade. The scale bar gives genomic distance in coalescent units, and is based on the amount of discordance in gene trees.

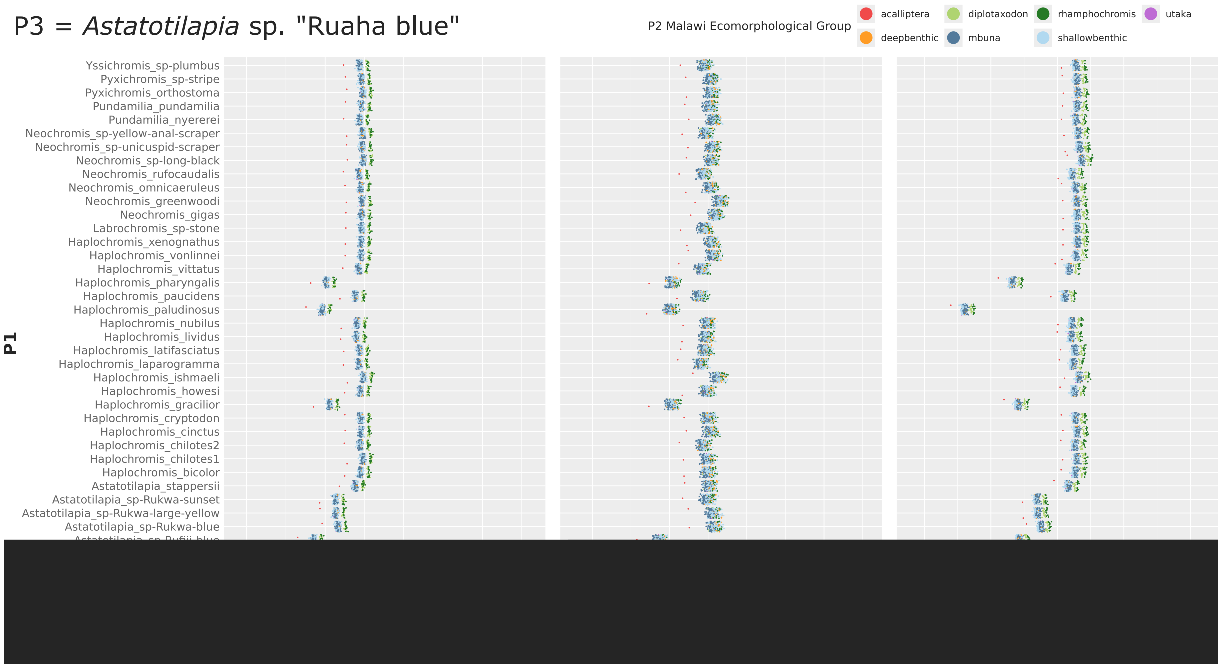

Supplementary Figure 5. D-statistic, Z-scores and f4-ratios of ABBA BABA trios with a significant D-statistic where P1 = Victoria clade species, P2 = Malawi species, and P3 = *Astatotilapia* sp. “Ruaha blue”. Trios are separated on the y-axis according to the P1 Victoria clade species and coloured according to the P2 Malawi ecomorphological group.

MOLECULAR ECOLOGY

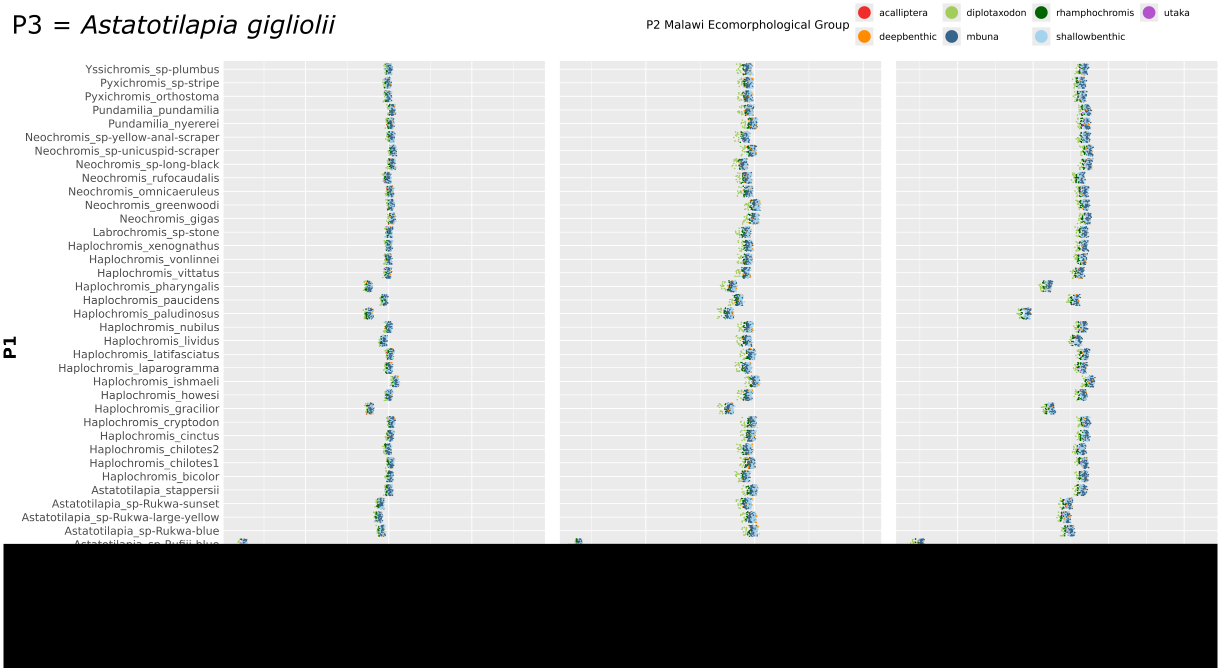

Supplementary Figure 6. D-statistic, Z-scores and f4-ratios of ABBA BABA trios with a significant D-statistic where P1 = Victoria clade species, P2 = Malawi species, and P3 = *Astatotilapia gigliolii*. Trios are separated on the y-axis according to the P1 Victoria clade species and coloured according to the P2 Malawi ecomorphological group.

MOLECULAR ECOLOGY

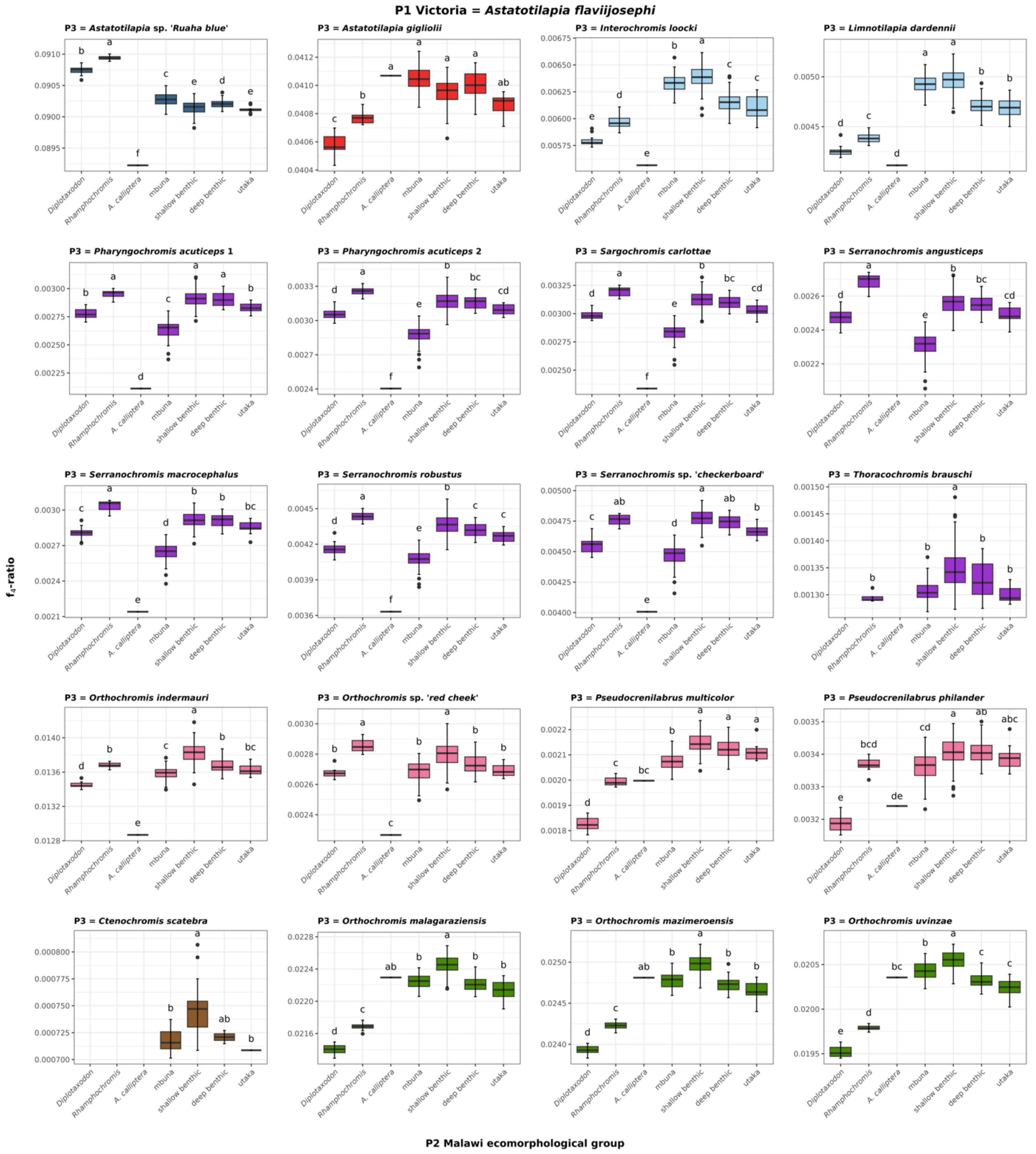

Supplementary Figure 7. f4-ratios of significant trios where species P1 = *Astatotilapia flaviijosephi* (Victoria group), P2 = Malawi, P3 = non-Malawi. Trios are separated into one of the seven Malawi ecomorphological groups (*Diplotaxodon*, *Rhamphochromis*, *Astatotilapia calliptera*, 'mbuna', shallow benthic, deep benthic, and 'utaka'). Boxplots are coloured according to the P3 non-Malawi group. Pairwise significant differences in the mean f4-ratio between groups (Tukey's HSD test, $p<0.05$) are indicated using letters to the side of each box; groups with different letters have significantly different means, whilst groups that share a letter do not.

MOLECULAR ECOLOGY

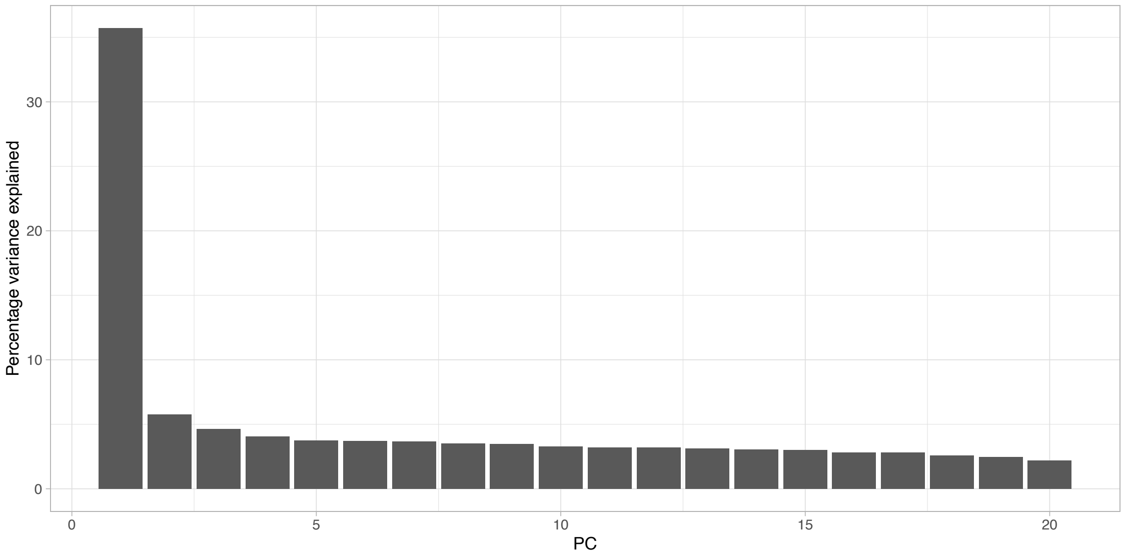

Supplementary Figure 8. Percentage of variance explained (PVE) by the first 20 principle components (PCs) in a principle component analysis (PCA) carried out on five *A. gigliolii*, four *A. calliptera* 'Rovuma', and 15 *A. calliptera* 'non-Rovuma' individuals.

MOLECULAR ECOLOGY

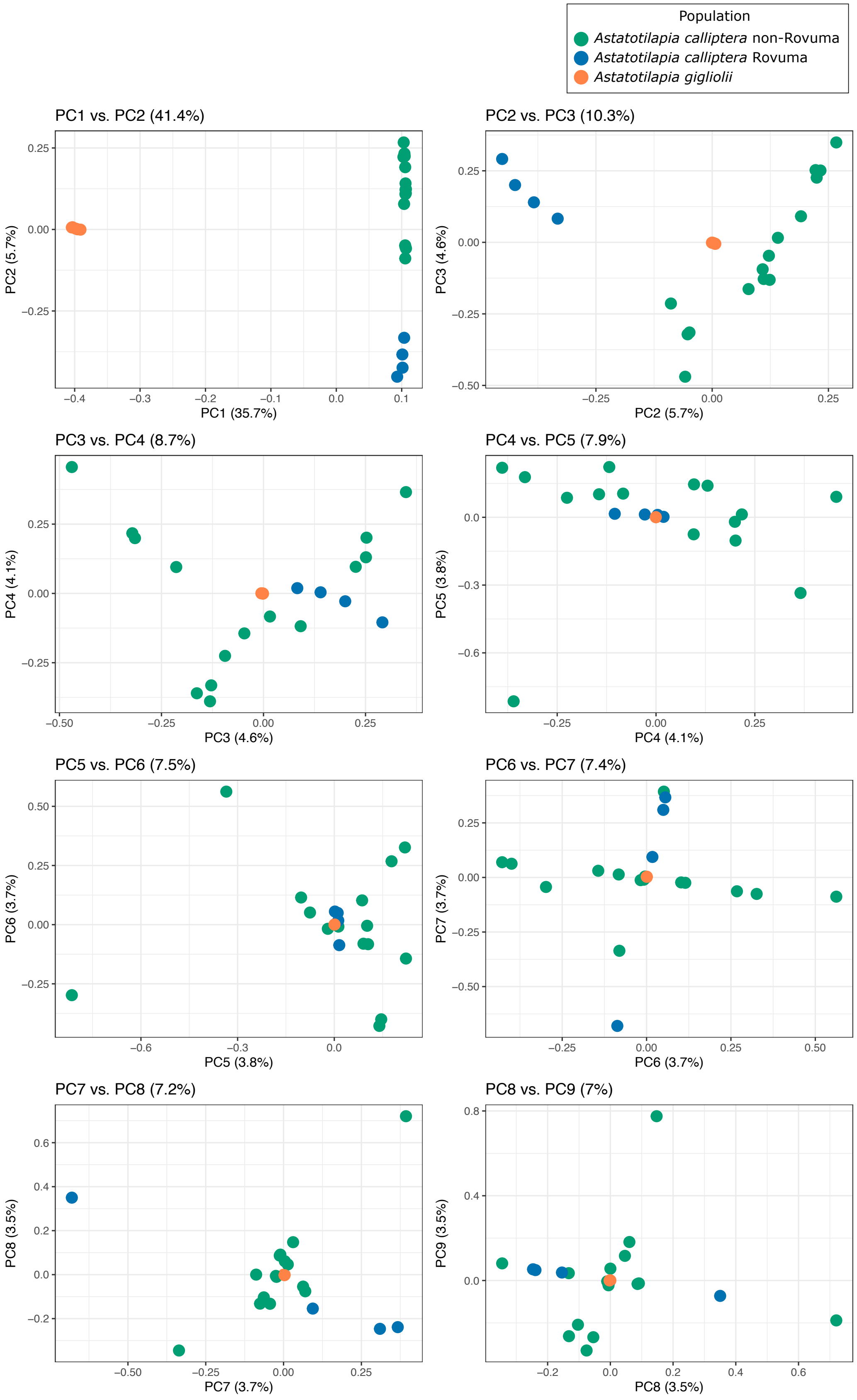

MOLECULAR ECOLOGY

Supplementary Figure 9. Principle component analysis (PCA) plot showing pairwise comparisons of principle components (PCs) from 1 to 9, performed on five *A. gigliolii* (orange), four *A. calliptera* 'Rovuma' (blue), and 15 *A. calliptera* 'non-Rovuma' (green) individuals. The percentage of variance explained (PVE) for each PC are denoted in brackets beside the PC, as well as the total across each comparison in each plot title.

MOLECULAR ECOLOGY

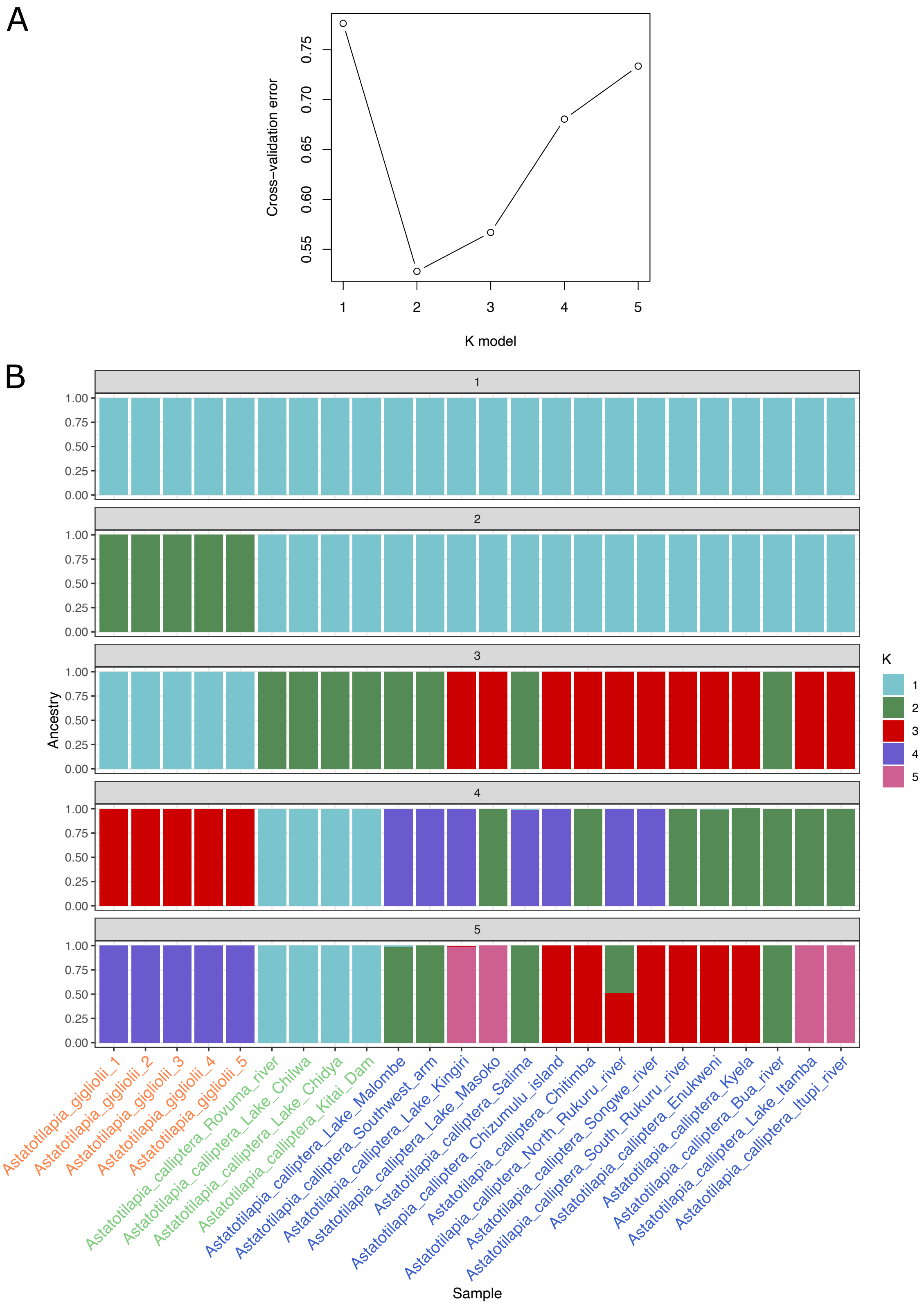

MOLECULAR ECOLOGY

Supplementary Figure 10. ADMIXTURE analysis performed on five *A. gigliolii* (orange), four *A. calliptera* 'Rovuma' (green), and 15 *A. calliptera* 'non-Rovuma' (blue) individuals. (a) Cross validation error values for each K model. (b) Ancestry proportions for K models 1-5 (best fitting model = 2).

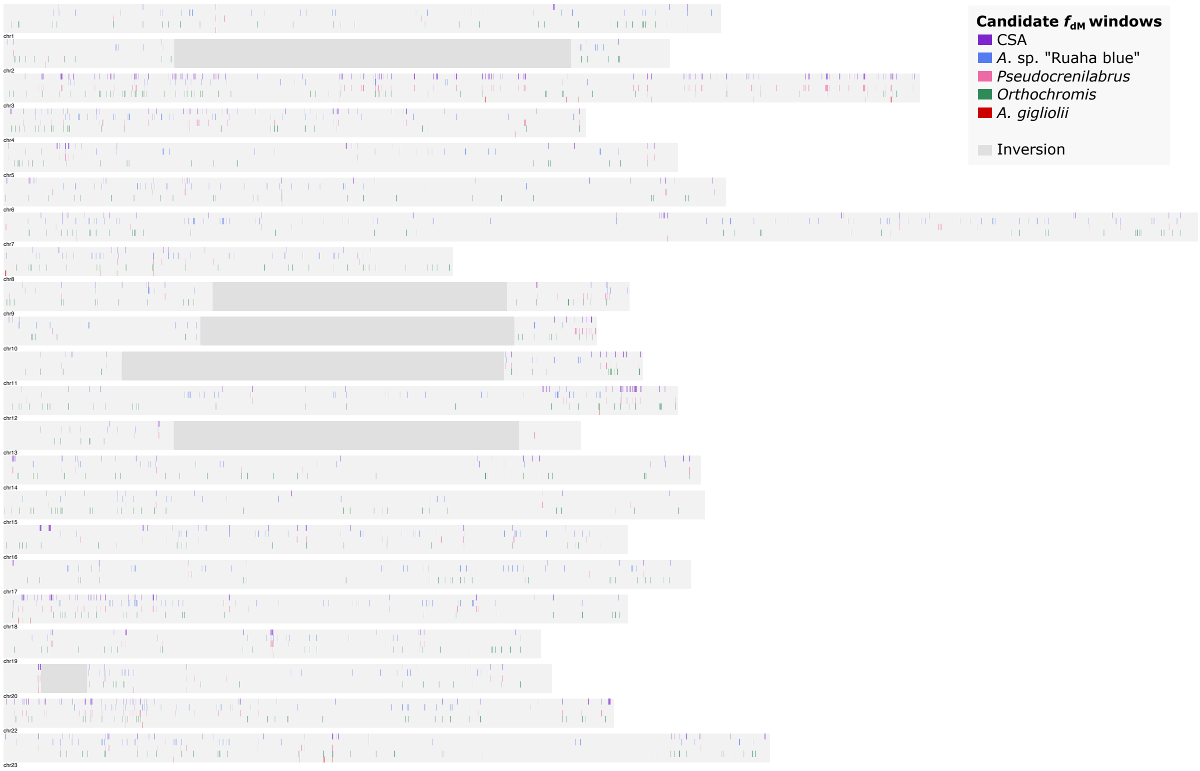

Supplementary Figure 11. Distribution of candidate introgressed f_{dM} windows across genome. Blocks are coloured according to which P3 group shows significant gene flow with Malawi; A. gigliolii (red), Orthochromis (green), Pseudocrenilabrus (pink), A. sp. "Ruaha blue" (blue), and CSA (purple).

MOLECULAR ECOLOGY

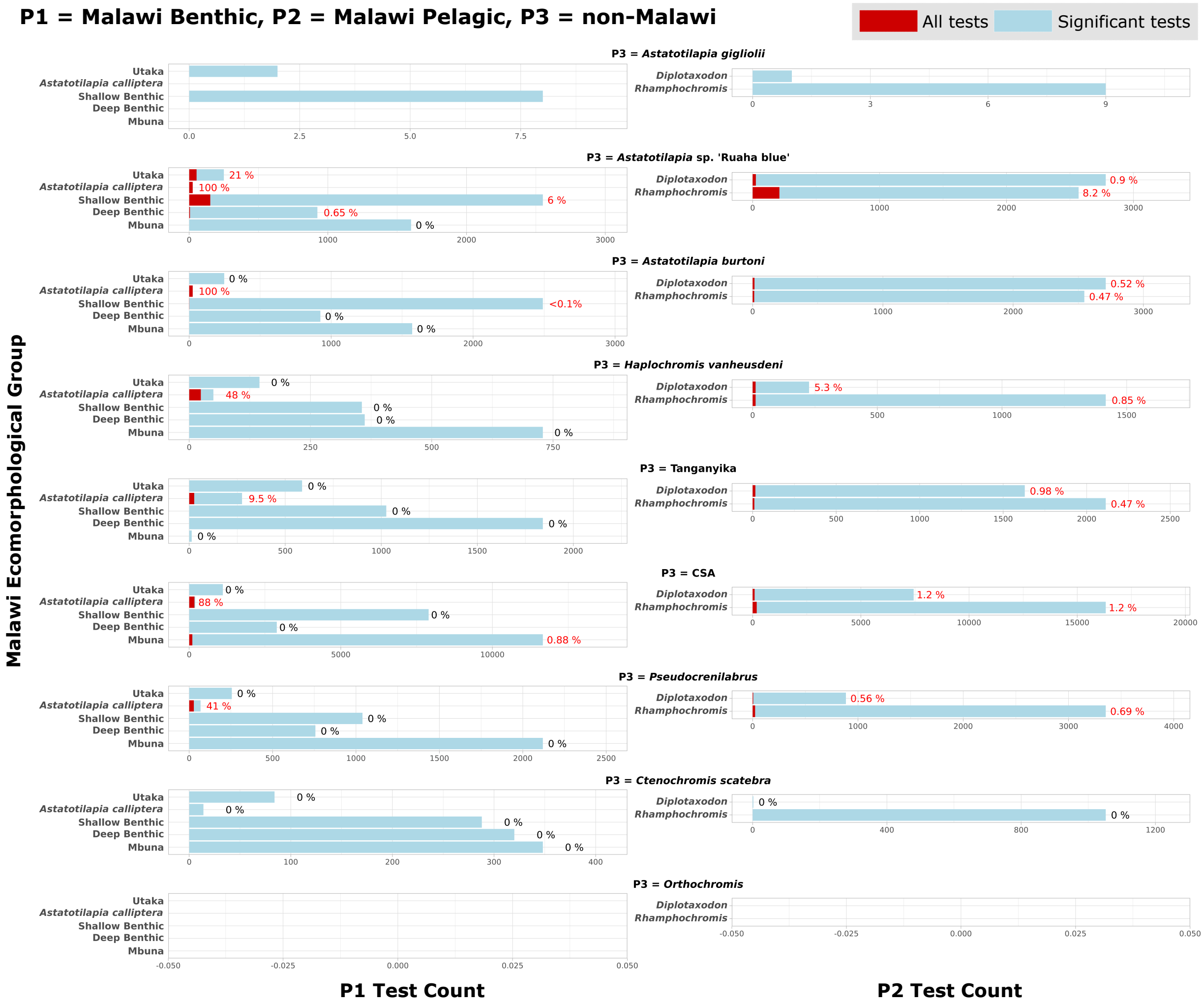

Supplementary Figure 12. Counts of ABBA BABA tests (all tests = blue, significant tests = red) where species P1 = Malawi benthic (i.e. from the *Astatotilapia calliptera*, 'utaka', shallow benthic, deep benthic, or 'mbuna' clades), P2 = Malawi pelagic (i.e. from the *Diplotaxodon* or *Rhamphochromis* clades), and P3 = non-Malawi. For each Malawi group as P1 or P2, the percentage of significant trios out of all trios is shown next to each bar. Percentages over 0% are shown in red.

MOLECULAR ECOLOGY

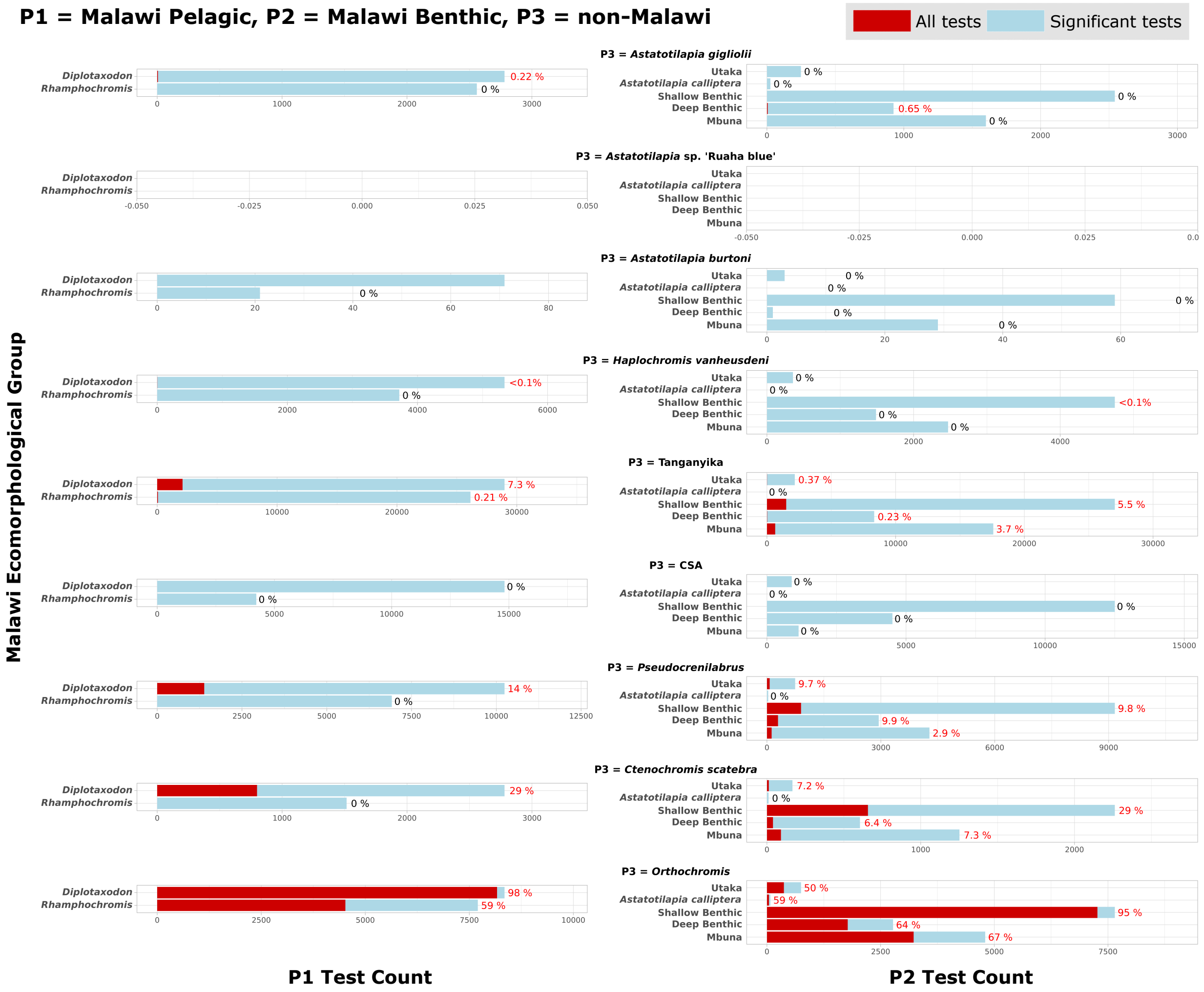

Supplementary Figure 13. Counts of ABBA BABA tests (all tests = blue, significant tests = red) where species P1 = Malawi pelagic (i.e. from the *Diplotaxodon* or *Rhamphochromis* clades), P2 = Malawi benthic (i.e. from the *Astatotilapia calliptera*, 'utaka', shallow benthic, deep benthic, or 'mbuna' clades), and P3 = non-Malawi. For each Malawi group as P1 or P2, the percentage of significant trios out of all trios is shown next to each bar. Percentages over 0% are shown in red.

MOLECULAR ECOLOGY

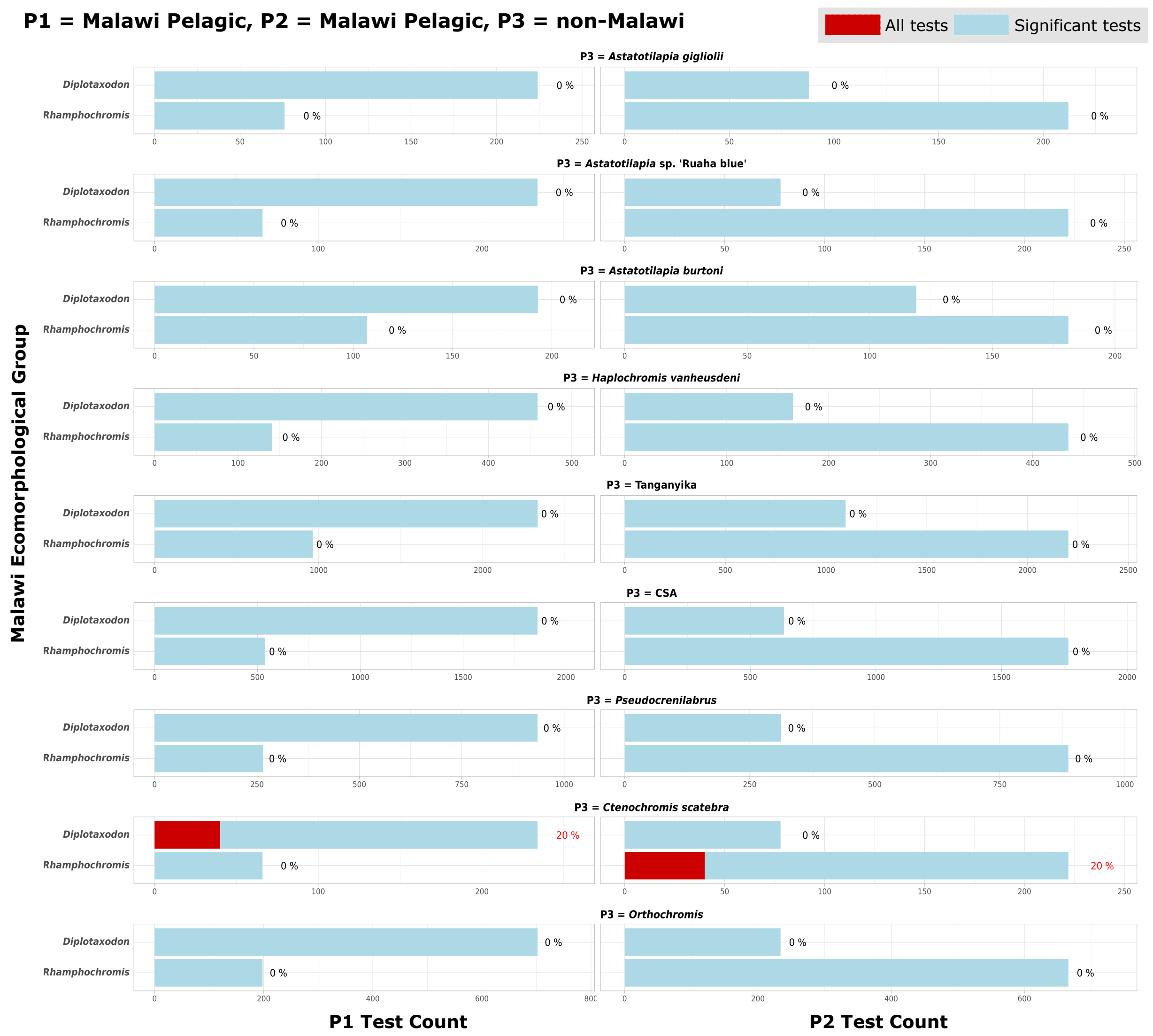

Supplementary Figure 14. Counts of ABBA BABA tests (all tests = blue, significant tests = red) where species P1 and P2 = Malawi pelagic (i.e. from the *Diplotaxodon* or *Rhamphochromis* clades), and P3 = non-Malawi. For each Malawi group as P1 or P2, the percentage of significant trios out of all trios is shown next to each bar. Percentages over 0% are shown in red.

MOLECULAR ECOLOGY

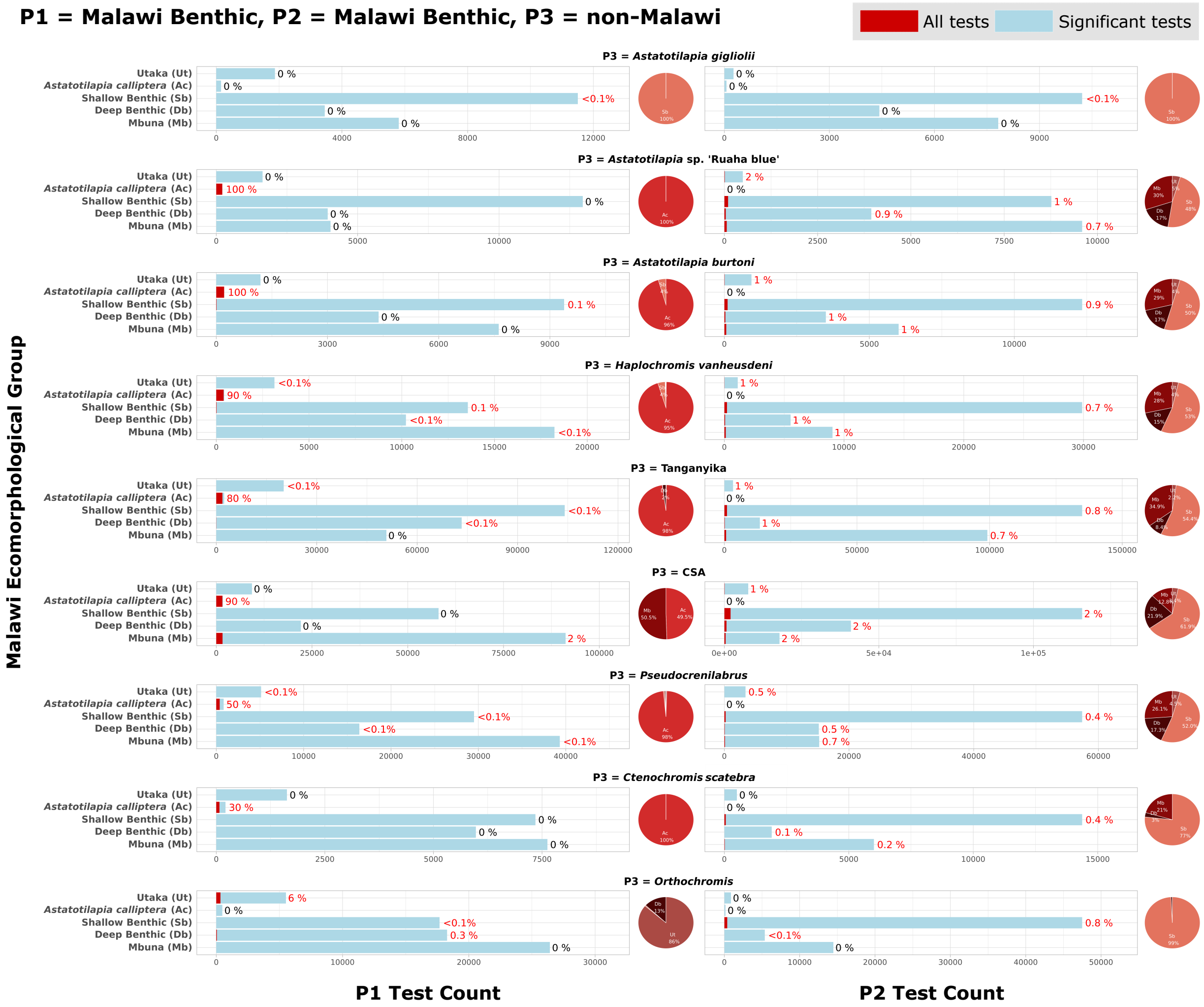

Supplementary Figure 15. Counts of ABBA BABA tests (all tests = blue, significant tests = red) where species P1 and P2 = Malawi benthic (i.e. from the *Astatotilapia calliptera*, 'utaka', shallow benthic, deep benthic, or 'mbuna' clades), and P3 = non-Malawi. For each Malawi group as P1 or P2, the percentage of significant trios out of all trios is shown next to each bar. Percentages over 0% are shown in red. For each P3 group, the share of significant trios across all Malawi groups is shown in a pie chart.
